# The effect of biogenic calcium phosphate nanoparticles on drought-stressed barley

**DOI:** 10.3389/fpls.2025.1660534

**Published:** 2025-12-04

**Authors:** Doaa E. Elsherif, Fatmah A. Safhi, Mai A. El-Esawy, Mohammed M. Mira, Nora M. Al Aboud, Esraa O. Razzaky

**Affiliations:** 1 Botany Department, Faculty of Science, Tanta University, Tanta, Egypt; 2 Department of Biology, College of Science, Princess Nourah bint Abdulrahman University, Riyadh, Saudi Arabia; 3 University Transfer, Portage College, Lac La Biche, AB, Canada; 4 Department of Biology, Faculty of Science, Umm Al-Qura University, Makkah, Saudi Arabia

**Keywords:** drought stress, barley, biogenic calcium phosphate nanoparticles, antioxidants, reactive oxygen species

## Abstract

Drought stress is one of abiotic stresses that significantly reduces agricultural yield annually. In response to drought, plants undergo several physiological and morphological changes like reduced transpiration and photosynthetic rate, disturbed osmotic adjustments, repressed root as well as shoot growth and overproduction of reactive oxygen species (ROS). In an experiment, a clay-sand mixture was placed in plastic pots containing barley seeds (Hordeum vulgare L., Giza 134), which were irrigated with 30% of the field capacity (FC). After one week, the pots were treated with different concentrations of biogenic calcium phosphate nanoparticles (CaPNPs) (25, 50 and 100 mg/L). Fourteen days later, the morpho-bio-physiological features were measured and documented. Applying 50 as well as 100 mg/L of CaPNPs on the well-watered leaves of barley plants increased shoot and root lengths, biomass, carbohydrates, non-enzymatic as well as enzymatic antioxidants, in addition to gene expression of superoxide dismutase (*HvSOD*) and catalase (*HvCAT*); such dosages mainly have been the optimal doses under normal conditions. Since calcium is a second messenger molecule, it can activate a variety of physiological signaling pathways when applied topically, mitigating the negative effects of drought stress on the development and metabolism of barley. Therefore, the application of CaPNPs yielded significant improvements across various plant functions despite drought stress conditions. Notably, there was an enhancement in growth parameters, osmo-protectants, and both cellular enzymatic and non-enzymatic antioxidants. At the molecular level, genes associated with betaine aldehyde dehydrogenase (*HvBADH1*), mitogen-activated protein kinases (*HvMAPK3*), showed provoked activity, particularly at a concentration of 25 mg/L. Furthermore, the treatment led to a decrease in ROS, as evidenced by reduced levels of malondialdehyde (MDA) and hydrogen peroxide. This reduction in ROS indicates an overall impediment of oxidative stress in the plants. Overall, the results of this study provide new insights into the molecular and physiological processes behind *H. vulgare*’s response to the optimal dose of biogenic CaPNPs, which is 50 and 100 mg/L in majority of the parameters in normal conditions and 25 mg/L under drought ones.

## 1 Introduction

By 2050, the world’s population is expected to reach 9.6 billion, requiring food production to increase by 70–100% to meet demand (Alabdallah et al., 2021). Yet, food production is increasingly threatened by climate change, abiotic stresses, and environmental degradation (Van Nguyen et al., 2022).

Climate change is a worldwide issue that has greatly affected agricultural productivity, posing risks to food security and supply. Projections indicate that the global average temperature could increase by 1.8 to 4°C by the year 2100 (Al-Khayri et al., 2023). As temperatures climb, the demand for irrigation water is anticipated to rise by 40% to 250% (Woznicki et al., 2015), especially in tropical areas facing inconsistent rainfall and frequent agricultural droughts. Water stress negatively affects photosynthesis, respiration, seed germination, nutrient uptake, and overall plant development (Shah et al., 2022; Duan et al., 2023; Hayat et al., 2023). It also causes oxidative damage through excessive reactive oxygen species (ROS), that damages DNA, causes lipid peroxidation (LPO), and denatures proteins, all of which impede productivity and cell growth (Hayat et al., 2023). Hormones, osmolyte buildup, and the activation of both non-enzymatic and enzymatic detoxifying systems are only a few of the numerous strategies used by plants in coping with such oxidative damage (Rasheed et al., 2022b). To ensure food security, improving drought tolerance in crops is therefore a global priority (Eckstein et al., 2021).

Barley (*Hordeum vulgare* L.) is a globally important cereal crop cultivated in temperate regions for both food and feed. It contributes significantly to human health through dietary fibers and is widely used in animal nutrition (Badea and Wijekoon, 2021). Although barley exhibits moderate drought tolerance, differences exist among genotypes; for example, the Giza 134 genotype has been identified as moderately tolerant under water-limited conditions (Mansour et al., 2017).

The effects of abiotic stresses on plants are a major challenge, prompting growing interest in strategies like the use of nanoparticles to reduce their harmful impacts—an area gaining attention in both agriculture and agricultural economics. Nanotechnology offers promising solutions for enhancing plant resilience under stress. Metal- and carbon-based nanoparticles (NPs) have shown a nutrient uptake improvement, photosynthesis enhancement, and antioxidant defense activation during drought stress in several crops (Naidu et al., 2023; Samynathan et al., 2023). As mentioned by Rezagholi et al. (2025) and Osundinakin et al. (2025), the antioxidant properties of nanoparticles contribute to enhancing the expression of crucial genes responsible for enzyme production under drought stress. These nanoparticles help alleviate the negative impacts of drought by directly or indirectly improving plant resilience, productivity, and traits related to growth.

Calcium phosphate nanoparticles (CaPNPs) are of growing interest because calcium plays a central role in plant signaling, enzyme activity, and stress response (Meier et al., 2020; Azeez et al., 2021). Recent studies suggest that CaPNPs can improve plant growth, water relations, and antioxidant activity (Badihi et al., 2021). During the last decade, these materials have raised a great interest in the development of macronutrient nano-fertilizers mainly due to their intrinsic composition in macronutrients (Ca, P) and their higher surface-to-volume ratio, enabling their functionalization (Carmona Fernández, 2022). Moreover, phosphorus stands out as a valuable element known for its role in nutrition and enhancing plant resistance to stresses, including drought. While research on CaPNPs’s effects on plant responses to various abiotic stresses is expanding, the specific mechanisms by which they influence plants under drought—regarding antioxidative status and the expression of genes—remain insufficiently understood.

Research on biogenic CaPNPs in plant stress tolerance is still emerging. While studies of chemical synthesis of CaPNPs have shown promising results in rice and snap beans on enhancing growth, the effects of biogenic CaPNPs as ecofriendly fertilizer on barley under drought stress remain largely unexplored (El-Ghany et al., 2021; Rasheed et al., 2022a). Hence, this study aims to evaluate the effect of novel approach in the form of foliar application of different concentrations of biogenic CaPNPs on barley genotype Giza 134 under or without drought stress. Alongside analyzing morpho-physiological responses and antioxidant activities, it also examines changes in the expression of genes seeking to provide new insights into nanoparticle-assisted stress mitigation in cereal crops. These findings could help in creating affordable methods to enhance drought tolerance in plants. We aim to investigate whether CaPNPs can ameliorate the harmful effects of drought stress in *H. vulgare*, in addition to understanding the underlying mechanisms which enable CaPNPs to combat such drastic effects imposed by water stress.

## 2 Materials and methods

### 2.1 Location of study and experiment

The experimental study was conducted at the sheltered plant garden of Botany Department, Faculty of Science, Tanta University, Egypt, according to the Randomized Complete Block Design. The experiment was conducted under controlled greenhouse conditions and experienced an average temperature of around 19°C (66°F), with a high temperature of about 25°C (78°F). The environmental growth conditions were 10 h photoperiod and ~65% relative humidity. According to Elshaboury and AlMetwaly (2023), Tanta city is the capital of El Gharbeya Governorate and is positioned at approximately 30° 47′28″N latitude and 30 ° 59′53″E longitude, with an elevation of about 10 meters above sea level. Tanta which is located in the central region of the Nile Delta in northern Egypt, is surrounded by fertile agricultural land. It sits on a flat, alluvial plain rich in black, nutrient-dense soil deposited by the Nile. The area’s geological makeup includes deep, well-developed soil layers, although distinct horizons are often missing, which places these soils within the Entisols category.

To assess the accurate dose of drought, in addition to the effective concentrations of CaPNPs, we first conducted preliminary experiment in November 2022, and we observed significant morphological and metabolic differences between the nano-particle treatments and the nano-drought treatments (such figures were demonstrated in the **Supplementary Material**). To confirm that this was a reproducible biological effect and not a one-time anomaly, we performed an independent repeat of the entire experiment. Therefore, the experiment was repeated in November 2023 using a new set of plants and under the same experimental conditions made in the previous season to verify the consistency of the obtained results. This new set of plants was treated, subjected to drought, harvested, and analyzed separately from the first cohort. The results from this second, independent experiment consistently confirmed the trends and statistical significance observed in the first run. Therefore, the experiment was performed twice in two different seasons with consistent results.

### 2.2 Calcium phosphate nanoparticles synthesis

Macroalgae *Jania rubens* was collected from Ras Gharib, located in the Gulf of Suez, Red Sea Governorate, Egypt. After collecting the algae, they were rinsed with distilled water, air-dried for 3 to 4 days, and then ground into a fine powder. Approximately 5 grams of this powder were mixed with 100 ml of distilled water and then centrifuged. Following the method described by El-Esawy et al. (2025) for CaPNPs synthesis, the supernatant was used in the process. Specifically, 1.5 ml of the aqueous *J. rubens* extract was mixed with 7.5 ml of 12.5 mM sodium phosphate (dibasic) solution and 7.5 ml of 12.5 mM calcium chloride, with continuous stirring for 10–15 minutes. The mixture was then sonicated for 30 minutes. The resulting CaPNPs were washed twice with ethanol and distilled water to remove impurities, followed by centrifugation at 6 xg for 10 minutes. Finally, the collected precipitate was dried in a vacuum oven at 60°C for 12 hours.

Before the foliar spray application, deionized water was utilized, and sonication for 15 min. was applied for the dispersion of the nanoparticles within the solution.

### 2.3 Plant materials

**Figure 1:**
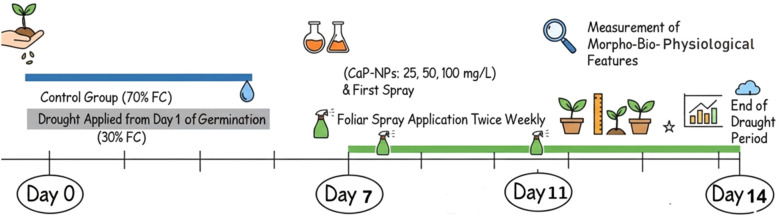
A Scheme illustrates the treatment timeline from cultivation till harvest.

Barley (*Hordeum vulgare*, L.) seeds were surface sterilized using 5% sodium hypochlorite for five minutes, then thoroughly rinsed with distilled water. A clay-sand mixture (2:1 w/w) was placed in 30 cm depth and 25 cm diameter pots containing barley seeds (Giza 134). The physiochemical properties of the soil used revealed the following concentrations: N = 0.7, P = 2.4, K = 34.3, Ca = 5.6, Mg = 23.2, Na = 6.1, and Cl = 6.69 mg/kg. The soil had a slightly neutral pH of 7.1 and an electrical conductivity (EC) of 3.5 mS/cm. Soil moisture was already calculated through Gravimetric method: Soil samples were saturated with water for 24–48 hours, allowing complete absorption, then drained to remove excess water. The gravimetric water content (WC) at field capacity (FC) was measured by weighing the soil before and after oven-drying at 105°C for 24 hours. Therefore, the drought conditions were applied to be 30% FC. In addition, the FC was not only estimated but also continuously monitored and maintained using a Capacitive Soil Moisture Sensor Module (v1.2, corrosion-resistant probe, DC 3.3–12 V, analog output, compatible with Arduino). This sensor allowed accurate detection of soil WC and ensured that the targeted FC was consistently controlled throughout the experiment. Tap water was used to irrigate the first group of pots, namely non-water stress (control, watered and maintained at 70% FC) until complete germination. Other pots (prone to severe water stress) were irrigated with 30% of the FC from day one of germination, which after one week of cultivation was subjected to foliar spraying of biogenic CaP-NPs twice weekly in 4 groups as follows: drought + 25 mg/L CaP-NPs, drought + 50 mg/L CaP-NPs, and drought + 100 mg/L CaPNPs. The same soil and watering levels were used for the initial germination and growth, for control and drought treatments. Fourteen days later, the morpho-bio-physiological features were measured and documented. The treatment timeline from cultivation till harvest is demonstrated in **Figure 1**.

### 2.4 Degree/level of drought stress

In the present study, drought intensity was imposed by maintaining FC at 30%, which is generally classified as severe drought stress. The intensity was further confirmed by plant-based indicators such as reduced WC. According to Ying et al. (2015), the drought levels are divided into three drought stress levels, namely non-water stress (control, watered and maintained at 75% field capacity), moderate water stress (MWS, watered and maintained at 50% field capacity) and severe water stress (SWS, watered and maintained at 30% field capacity). Typically, a combination of methods: morphological (reduced growth), physiological (Pro and MDA), in addition to soil-based (Gravimetric method), were used to accurately determine drought level.

### 2.5 Measurement of hydrogen peroxide

Hydrogen peroxide (H_2_O_2_) concentration was quantified spectrophotometrically using the method of Velikova et al. (2000). Briefly, 200 mg of leaf tissue was homogenized in an ice bath with 1 mL of 0.1% (w/v) trichloroacetic acid (TCA). The homogenate was centrifuged, and the supernatant was used for the assay. The reaction mixture contained the supernatant, 10 mM potassium phosphate buffer (pH 5.8), and 1 M potassium iodide (KI). The absorbance of the resulting mixture was measured at 390 nm. The H_2_O_2_ concentration was determined using a standard curve and is expressed as µmol per gram fresh weight (µmol/g f.wt.).

### 2.6 Lipid peroxidation

Malondialdehyde (MDA) levels were measured using the method outlined by Heath and Packer (1968). Fresh tissues (0.25 g) were homogenized in 5 ml of 0.1% (w/v) trichloroacetic acid, centrifuged at 15 xg for 10 min. The supernatant (0.5 ml) was added to 2 ml thiobarbituric acid (TBA) in 20% (w/v) TCA. Cooling was applied to the reaction mixture, followed by heating to 95°C for 30 minutes. The absorbance was recorded at both 532 and 600 nm.

### 2.7 Total antioxidant capacity

Phosphomolybdenum technique for measuring TAC was measured in accordance with Prieto et al. (1999), where sulfuric acid (0.6 M), ammonium molybdate tetrahydrate (4 mM), and sodium phosphate dibasic solution (28 mM) were added to create the TAC reagent. TAC reagent was combined with ethanolic extract and cooked for ninety minutes. Sample absorbance was measured at 765 nm after cooling.

### 2.8 Carbohydrates

Fresh plant tissues stored in liquid nitrogen were dried at 60°C until reaching a constant weight. Tissues (0.1 g) were homogenized in 80% ethanol and then incubated at 80°C for about 30 minutes. After centrifugation at 13 xg for 10 minutes, the supernatant was transferred to a new tube, vacuum dried and resuspended in water. 1 ml of the supernatant was combined with 0.5 ml of 5% phenol solution, followed by the rapid addition of 2.5 ml of concentrated sulfuric acid, incubated at room temperature for 10 minutes, then placed in a water bath at 25–30°C for 20 minutes to develop color. The absorbance was measured at 490 nm using a spectrophotometer, with distilled water as the blank (Masuko et al., 2005).

### 2.9 Proline

The amount of proline (Pro) in the dried leaf powder was determined by dissolving it in 3% sulfosalicylic acid according to Bates et al. (1973) with ninhydrin reagent. Following extraction with toluene, the resulting chromophore was monitored at 520 nm. A calibration curve was created for Pro, and the concentration was determined in milligrams per kilogram of dry matter.

### 2.10 Water content

Barley fresh leaves were ground and centrifuged at 5 xg. The supernatant was analyzed using a 5500 Vapor Pressure Osmometer (Wescor). The WC was calculated using the formula WC = [(fresh weight - dry weight)/(fresh weight)] x 100 (Jin et al., 2017). Dry weight was calculated oven-drying samples at 105°C for 24 hours.

### 2.11 Determination of phenolics and flavonoids content

Fresh leaves were pulverized into fine powder after being dried. At room temperature, tissues (0.1 and 0.5 g) were homogenized and incubated for 30 minutes in 70% acetone and then centrifuged at 4 xg. Phenolic content was quantified using Folin Ciocalteu reagent. The extract was mixed with Folin-Ciocalteu reagent (1:1) (V: V). Sodium carbonate solution 7.5% was added (2V) after 5 minutes. After 2 h, the absorbance was measured at 725 nm (Ainsworth and Gillespie, 2007). 100 µl of the same extract was mixed with 4 ml of distilled water for the flavonoid assay, and 0.3 ml of 5% sodium nitrite was added after that. Five minutes later, 0.3 ml of 10% aluminum chloride was added. 2 ml of 1 M sodium hydroxide was added to the mixture after 5 minutes. The absorbance was measured against a blank at 510 nm. Catechin was used for the calibration curve (Aynalem and Kitaw, 2023).

### 2.12 Determination of glutathione

Glutathione (GSH) content was determined following the method of Anderson (1985). 0.1 g of fresh leaf tissue was homogenized in 5 ml of 3% (w/v) sulfosalicylic acid and centrifuged the mixture at 10 xg for 10 minutes. The reaction mixture consisted of 0.5 ml tissue extract, 0.5 ml of 0.5 mM potassium phosphate buffer (pH 7.0), and 50 µl of 3 mM 5,5-dithio-bis-2-nitrobenzoic acid (DTNB). The absorbance was measured at 412 nm and calculated the GSH content using a standard GSH curve.

### 2.13 Measurement of polyphenol oxidase, phenylalanine, and peroxidase activity

Fresh tissues (0.5 g) were immediately stored in liquid nitrogen and then homogenized in 5 mL of 0.1 M phosphate buffer (pH 6.5) with 1% polyvinylpyrrolidone (PVP). After centrifugation at 10 xg for 15 minutes at 4°C, the supernatant was added to a reaction mixture of 10 mM catechol in 0.1 M phosphate buffer (pH 6.5). PPO activity was reported according to Kumar and Khan (1982), the increase in absorbance at 420 nm over 1 minute. PAL activity was assayed according to Şirin et al. (2016) method. In a reaction, mixture contained enzyme extract, 100 mM Tris-HCl (pH 8.8), 40 mM l-phenylalanine, detecting the trans-cinnamic acid formation at 290 nm. After incubating at room temperature for half an hour, the reaction was stopped by adding 4M HCl and PAL specfic activity was expressed as µM/g.f.wt. min -1. The same supernatant was utilized to measure POD activity. the enzyme extract was added to a reaction mixture of 10 mM guaiacol and 10 mM H_2_O_2_ in 0.1 M phosphate buffer (pH 6.5. The increase in absorbance was reported at 470 nm over 1 minute.

### 2.14 Expression studies

RNA extraction was carried out using RNeasy Plant Mini Kit according to the instructions (Qiagen). cDNA was created using the High-Capacity cDNA Reverse Transcription Kit (Applied Biosystems). Quantitative RT-PCR was performed exactly as described by Mira et al. (2016). The relative gene expression level was analyzed with the 2^−ΔΔCT^ method (Livak and Schmittgen, 2001) using *Actin2* as the reference gene. The primers are listed in **Supplementary Table 1**.

### 2.15 Statistical analysis

All results presented are means of three biological replicates, each consisting of at least 4 plants. Analysis of variance of the data was conducted using the GLMMIX procedure (SAS Institute, 2005) of SAS University Edition Version 9.04.01. Treatment means were compared using the Tukey test (ɑ = 0.05). Pearson’s correlation analysis was conducted to evaluate relationships between growth parameters, antioxidant systems, and phytochemical compounds.

## 3 Results

### 3.1 Growth characteristics

**Figure 2:**
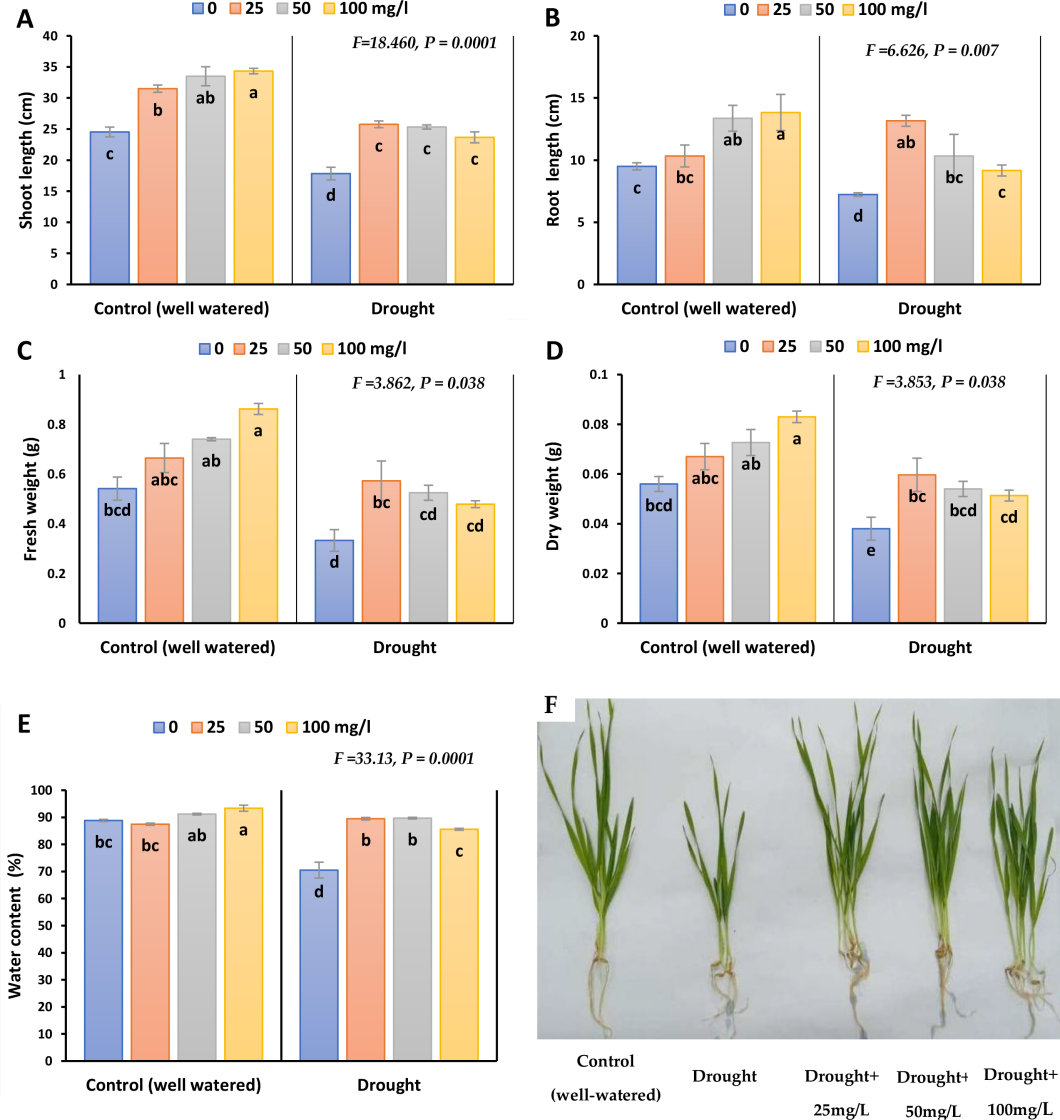
Growth parameters of barley plants treated with 25, 50 and 100 mg/L CaPNPs under control (well-watered) or (FC 30%) in 2023. **(A)** Shoot length, **(B)** Root length, **(C)** Fresh weight, **(D)** Dry weight, **(E)** Water content, **(F)** Representative image of barley plants under control and drought conditions with various CaPNP treatments. Values represent mean ± SD (n=3). Different letters indicate significant differences at p < 0.05 (Tukey’s).

**Figure 2**; **Supplementary Figure 1**. illustrates that drought stress significantly reduced growth parameters (shoot length, root length, fresh weight and dry weight of the plant). The reduction percentages were recorded as 31.2, 24.2, 36, and 31% respectively in 2023, whilst as 36.8, 24, 30.9 and 26% in 2022. The same trend was observed in the drought-treated plants sprayed with different concentrations of CaPNPs, each compared to their control. Nonetheless, non-sprayed stressed plants showed the lowest rate of growth parameters compared to stressed ones treated with CaPNPs concentrations. In non-stressed plants, adding CaPNPs enhanced shoot height, fresh weight and dry weight in proportion with their concentration and compared to their control, especially 100 mg/L by 40, 59 and 48% in 2023, whilst by 49, 52 and 43% in 2022, respectively. On the other hand, stressed plants treated with 25 mg/L recorded the highest rate of growth parameters including shoot height, root length, fresh and dry weights, reflecting its alleviatory effect. Also, the application of different concentrations of CaPNPs (25, 50 and 100 mg/L) to the water stressed plants promoted WC compared to drought stress.

### 3.2 Stress biomarkers

**Figure 3:**
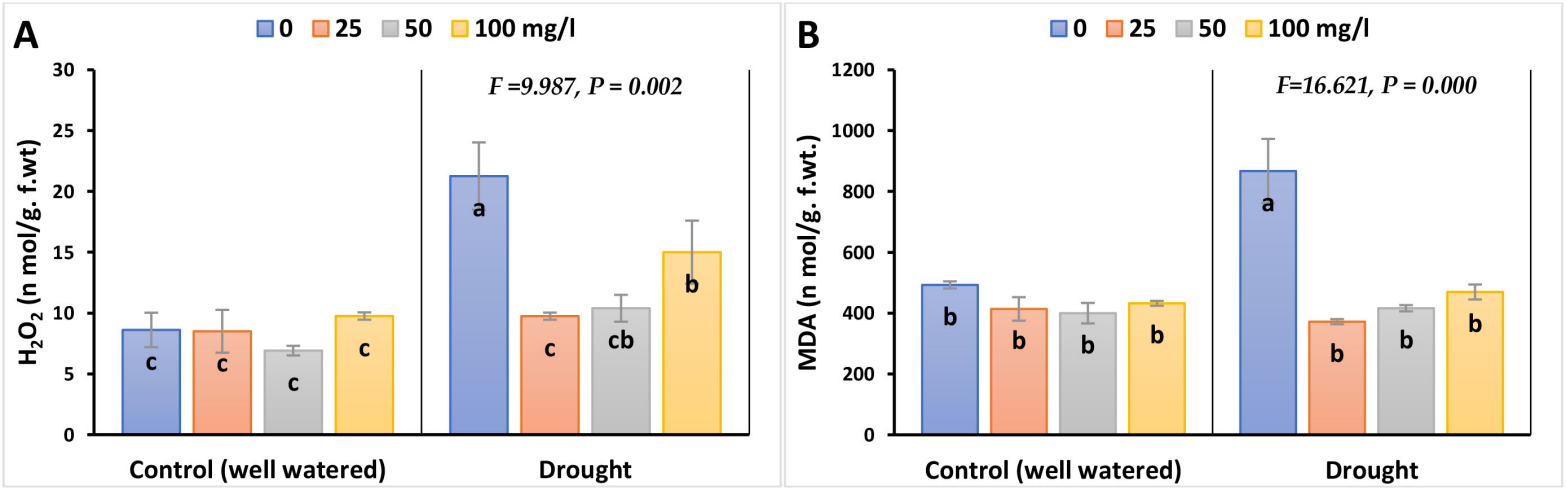
Effects of calcium phosphate nanoparticles (CaPNPs) on stress biomarkers in barley plants under well-watered (control) and drought stress (FC 30%) conditions in 2023. The figure presents the effects of various concentrations of CaPNPs (25, 50, and 100 mg/L) on two key stress indicators: **(A)** H_2_O_2_ (Hydrogen Peroxide) and **(B)** MDA (Malondialdehyde): The product of lipid peroxidation. Values represent mean ± SD (n=3). Different letters indicate significant differences at p < 0.05 (Tukey’s).

MDA (as a measure of LPO) and H_2_O_2_ (as a measure of oxidative stress) were significantly induced in barley leaves exposed to drought stress, compared to their control values, as illustrated in **Figure 3**; **Supplementary Figure 2**. The highest difference was proved to be between the stressed barley plants and their controls without adding nanoparticles by 76 and 147% in 2023, whilst by 96 and 136% in 2022, respectively. Interestingly, the results with CaPNPs revealed a discernible recovery impact and lessened the stress biomarker levels in drought-stressed leaves to reach values close to the non-stressed control, particularly the lowest dose (25 mg/L). The percentage differences that represent the tolerance for MDA and H_2_O_2_ were as follows: 57 and 52% in 2023, whilst 60 and 56% in 2022, each compared to their drought treatment.

### 3.3 Total antioxidant activity

**Figure 4:**
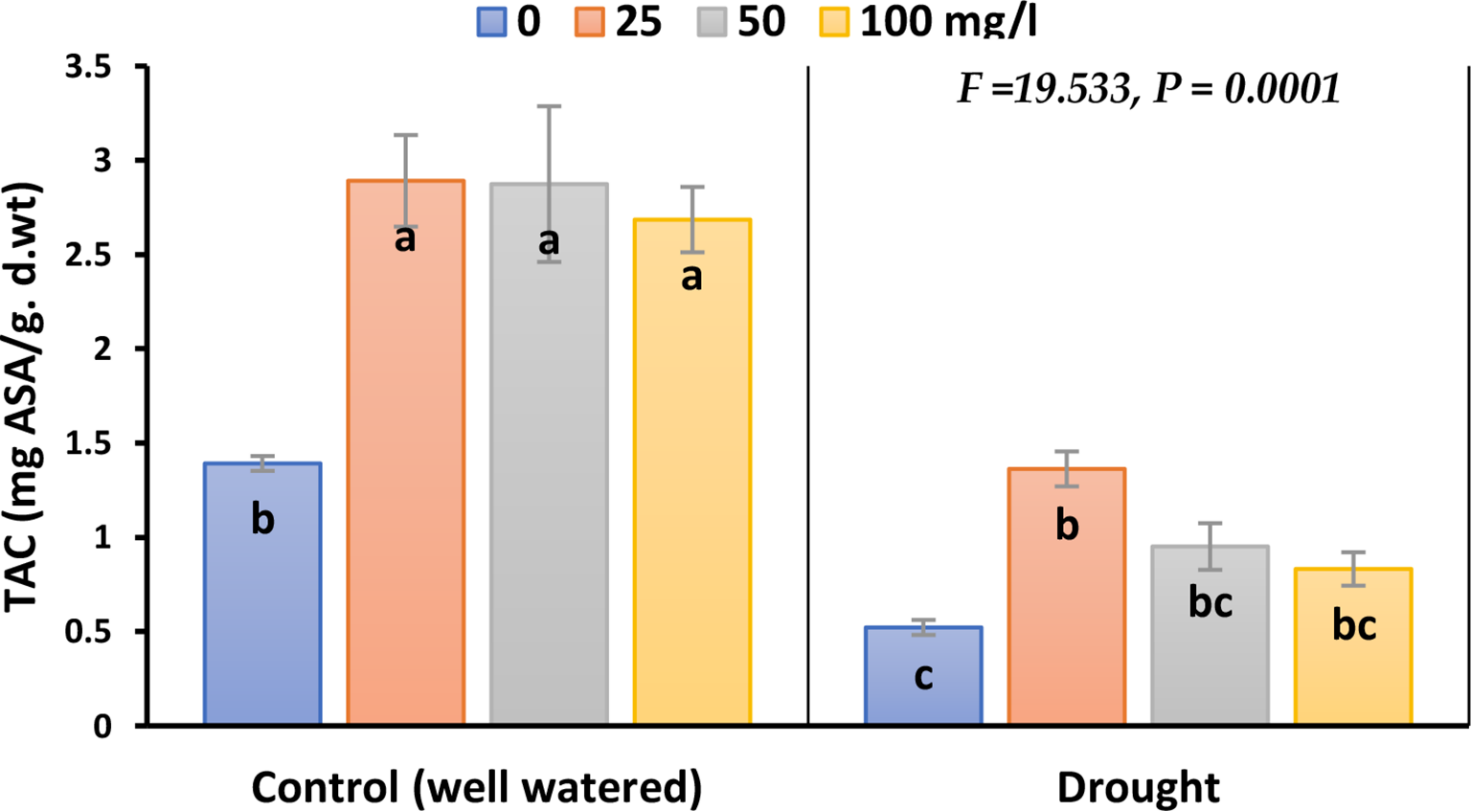
Total antioxidant capacity (TAC) of barley plants treated with 25, 50 and 100 mg/L CaPNPs under control (well-watered) or (FC 30%) in 2023. Values represent mean ± SD (n=3). Different letters indicate significant differences at p < 0.05 (Tukey’s).

The inhibitory effect of drought on TAC in plants treated with CaPNPs compared to its control was significant; the inhibition percentages were 63% in 2023, whilst 79% in 2022 (**Figure 4**; **Supplementary Figure 3**). Nevertheless, stressed and non-stressed plants recorded a remarkable elevation in TAC level upon CaPNPs treatments.

### 3.4 Osmoprotectants

**Figure 5:**
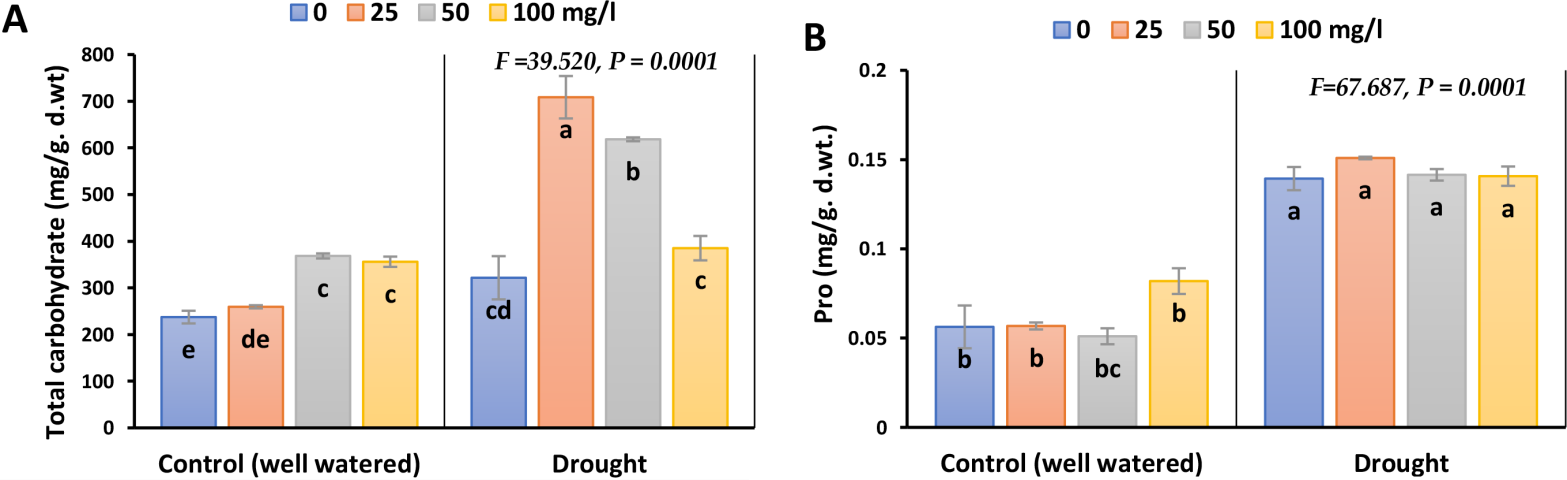
Osmolytes accumulation in barley plants treated with 25, 50 and 100 mg/L CaPNPs under control (well-watered) or (FC 30%) in 2023. **(A)** Total carbohydrate and **(B)** Proline. Values represent mean ± SD (n=3). Different letters indicate significant differences at p < 0.05 (Tukey’s).

Pro and carbohydrates were markedly induced under drought stress by 180 and 35.5% in 2023, whilst 122 and 53% in 2022, respectively (**Figure 5**; **Supplementary Figure 4**). During drought conditions, application of CaPNPs elevated total carbohydrates accumulation significantly, whilst the rate of enhancement in Pro was insignificant in both seasons.

### 3.5 Non-enzymatic antioxidant molecules

**Figure 6:**
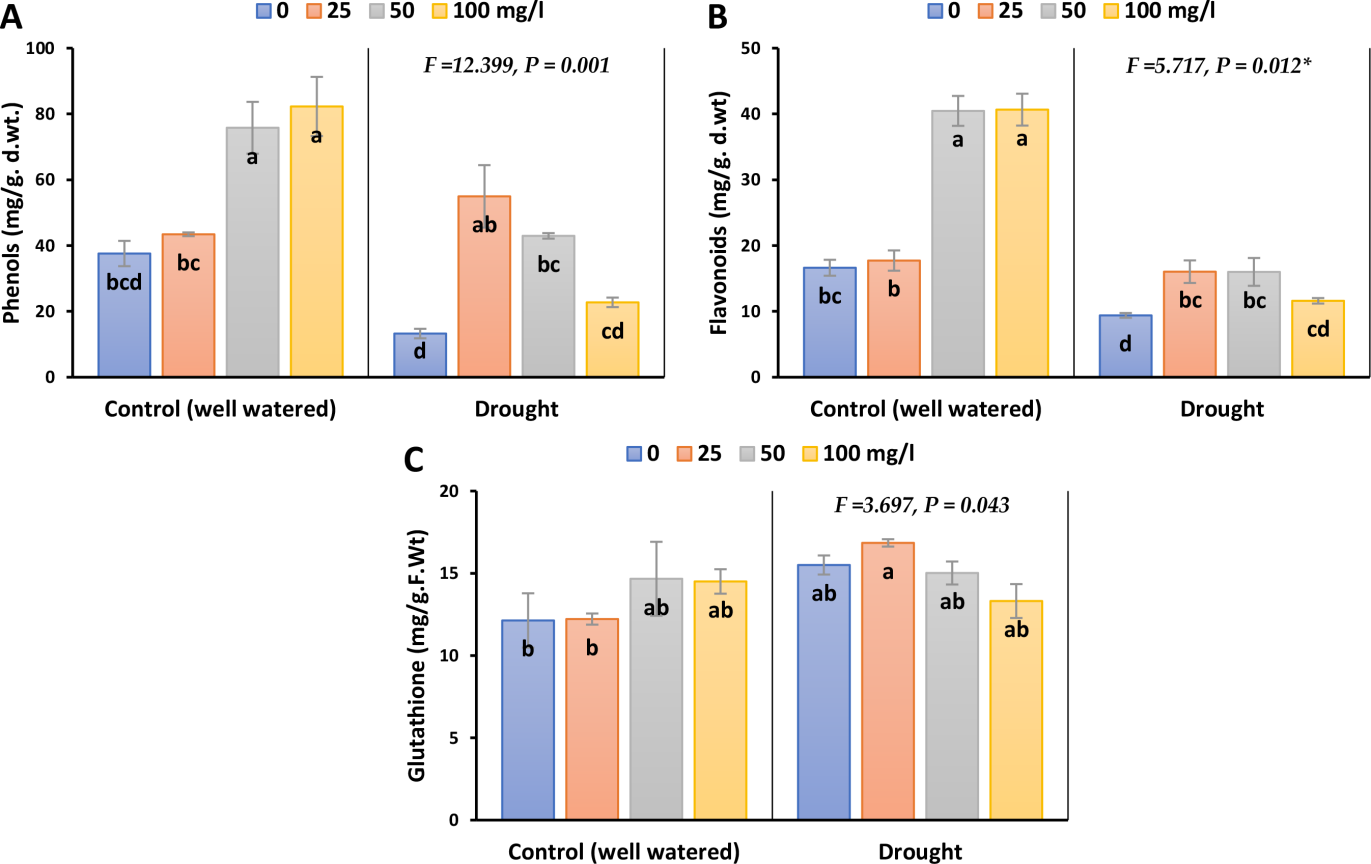
Non-enzymatic antioxidants of barley plants treated with 25, 50 and 100 mg/L CaPNPs under control (well-watered) or (FC 30%) in 2023. **(A)** Total phenolic contents. **(B)** Total Flavonoids, **(C)** Reduced Glutathione. Values represent mean ± SD (n=3). Different letters indicate significant differences at p < 0.05 (Tukey’s).

Non-enzymatic antioxidant compounds such as total phenolic contents, total flavonoid contents and reduced glutathione (GSH), in barley leaves showed significant variations in response to experimental treatments as demonstrated in **Figure 6**; **Supplementary Figure 5**. Drought stress markedly reduced total phenolic content (TPC) and total flavonoid content (TFC) by 65 and 43% in 2023, whilst by 79 and 36% in 2022, respectively. However, spraying with different concentrations of CaPNPs restored the level of TFC to values close to that of the control and exceeded the control under the application of 25 mg/L for TPC by 46.7% in 2023 and 54.6% in 2022. On the other hand, drought stress led to significant accumulation in GSH in the leaves by 23% in 2023 and 32% in 2022. The initial concentration (25 mg/L) enhanced GSH content during drought stress, with reference to its control.

### 3.6 Antioxidant enzyme activities

**Figure 7:**
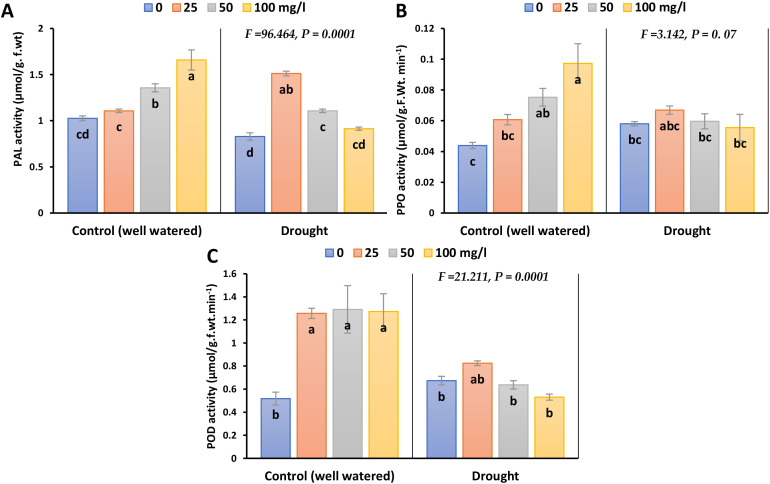
Activities of antioxidant enzymes of barley plants treated with 25, 50 and 100 mg/L CaPNPs under control (well-watered) or (FC 30%) in 2023. **(A)** Phenyl alanine activity, **(B)** PPO **(C)** POD activity. Values represent mean ± SD (n=3). Different letters indicate significant differences at p < 0.05 (Tukey’s).

Exposure to drought caused a reduction in Phenylalanine (PAL) activity, by 23.2% in 2023 and 28.9% in 2022 compared to their control (**Figure 7**; **Supplementary Figure 6**). Different doses of CaPNPs especially 25 mg/L, triggered a recovery response and markedly enhanced the activity of such enzyme relative to control by 53% in 2023 and 61.2% in 2022. Notably, the activities of Polyphenol Oxidase (PPO) and Peroxidase (POD) slightly increased under drought conditions. Nevertheless, the application of CaPNPs restored POD activity to levels like those recorded under control conditions.

### 3.7 Gene expression

**Figure 8:**
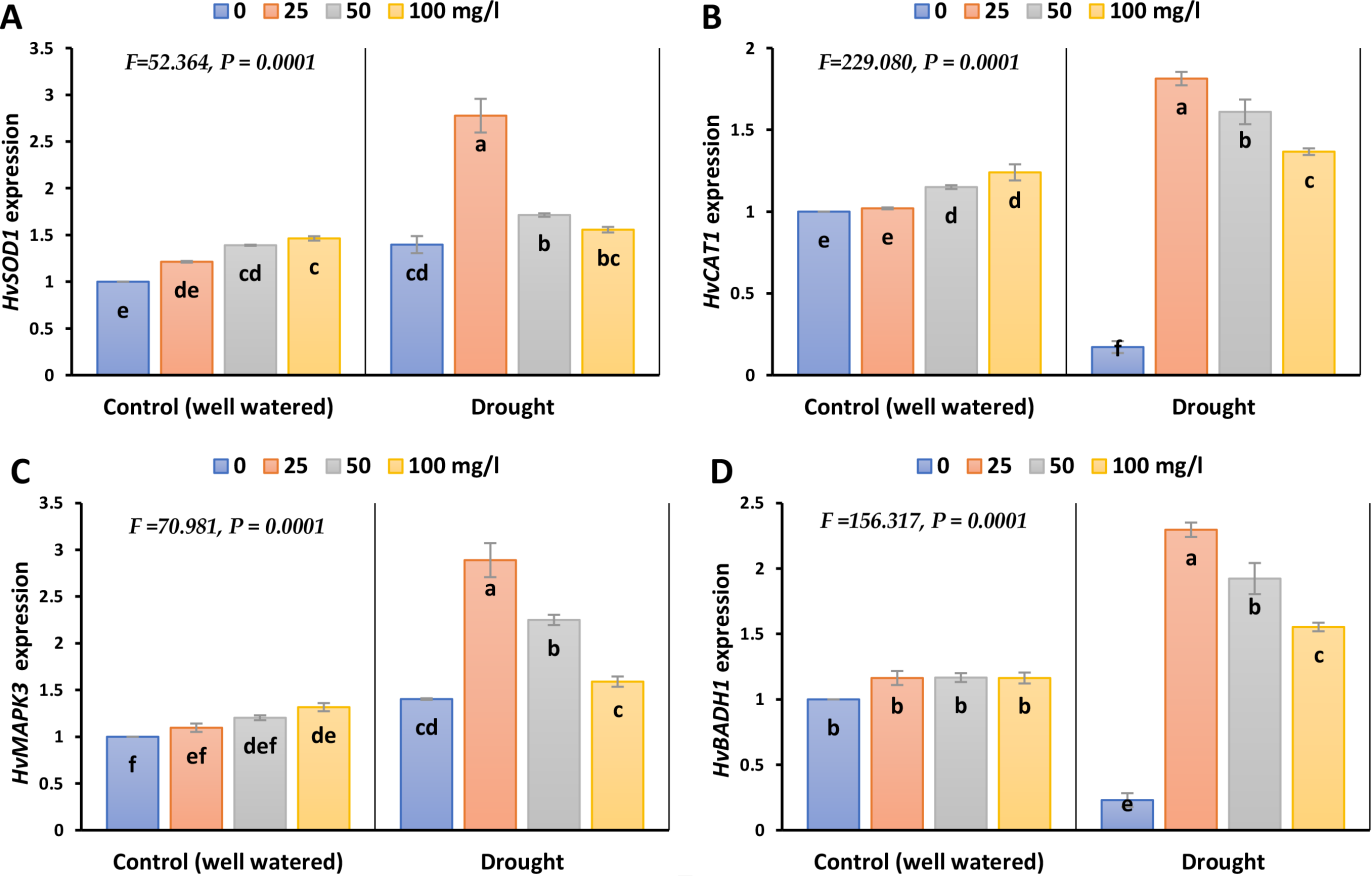
Gene expression (A. *HvSOD1* (*Superoxide Dismutase)*, **(B)***HvCAT1* (*Catalase*), **(C)***HvMAPK3* (*Mitogen Activated Protein Kinase*) and **(D)***HvBADH1* (*Betaine Aldehyde Dehydrogenase*) of barley plants treated with 25, 50 and 100 mg/L CaPNPs under control (well-watered) or (FC 30%) in 2023. Values represent mean ± SD (n=3). Different letters indicate significant differences at p < 0.05 (Tukey’s).

The expression levels of *HvSOD1* and *HvMAPK3* increased by 36 and 40% during drought stress, whilst the expression levels of *HvCAT1* and *HvBADH1* decreased by 83 and 77%, each compared to its control (**Figure 8**; **Supplementary Figure 7**). The different doses of CaPNPs triggered a recovery response and markedly induced the expression levels of *HvCAT1*, *HvSOD1*, *HvBADH1* and *HvMAPK3*.

### 3.8 Proposed model of CaPNPs effects on plant physiological and biochemical responses

**Figure 9:**
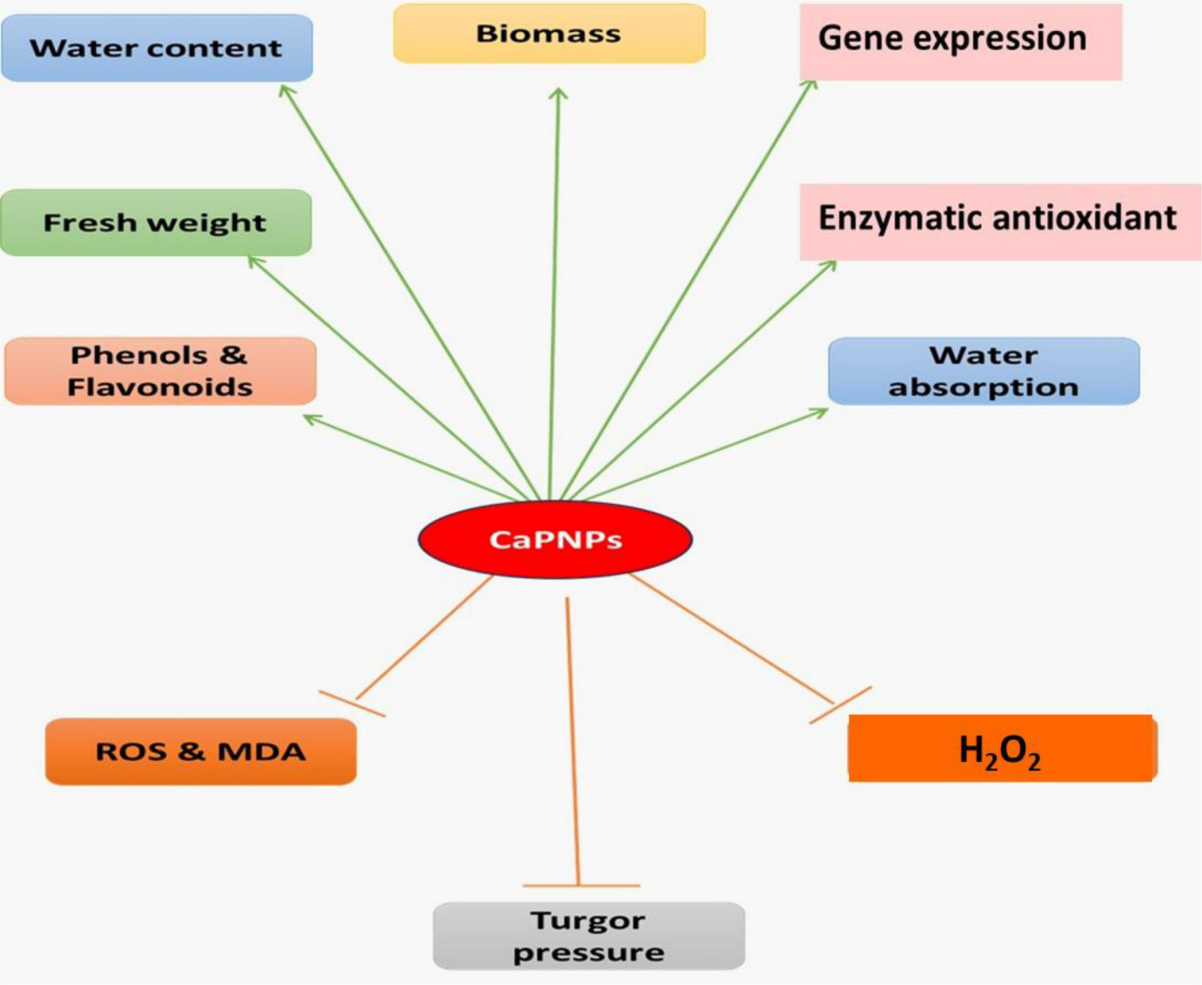
Schematic representation of the effects of calcium phosphate nanoparticles (CaPNPs) on various physiological and biochemical parameters in plants. Green arrows indicate positive effects, including increased biomass, water absorption, fresh weight, water content (WC), phenol & flavonoid accumulation, enzymatic antioxidants and gene expression. Orange lines represent negative effects, showing a reduction in reactive oxygen species (ROS) and malondialdehyde (MDA) levels, as well as H_2_O_2_. Dashed orange lines indicate potential impacts on turgor pressure.

**Figure 9** highlights the potential of CaPNPs to alleviate drought stress in barely in terms of growth characteristics, antioxidative modulation, in addition to molecular approaches.

### 3.9 Pearson correlation coefficient

**Figure 10:**
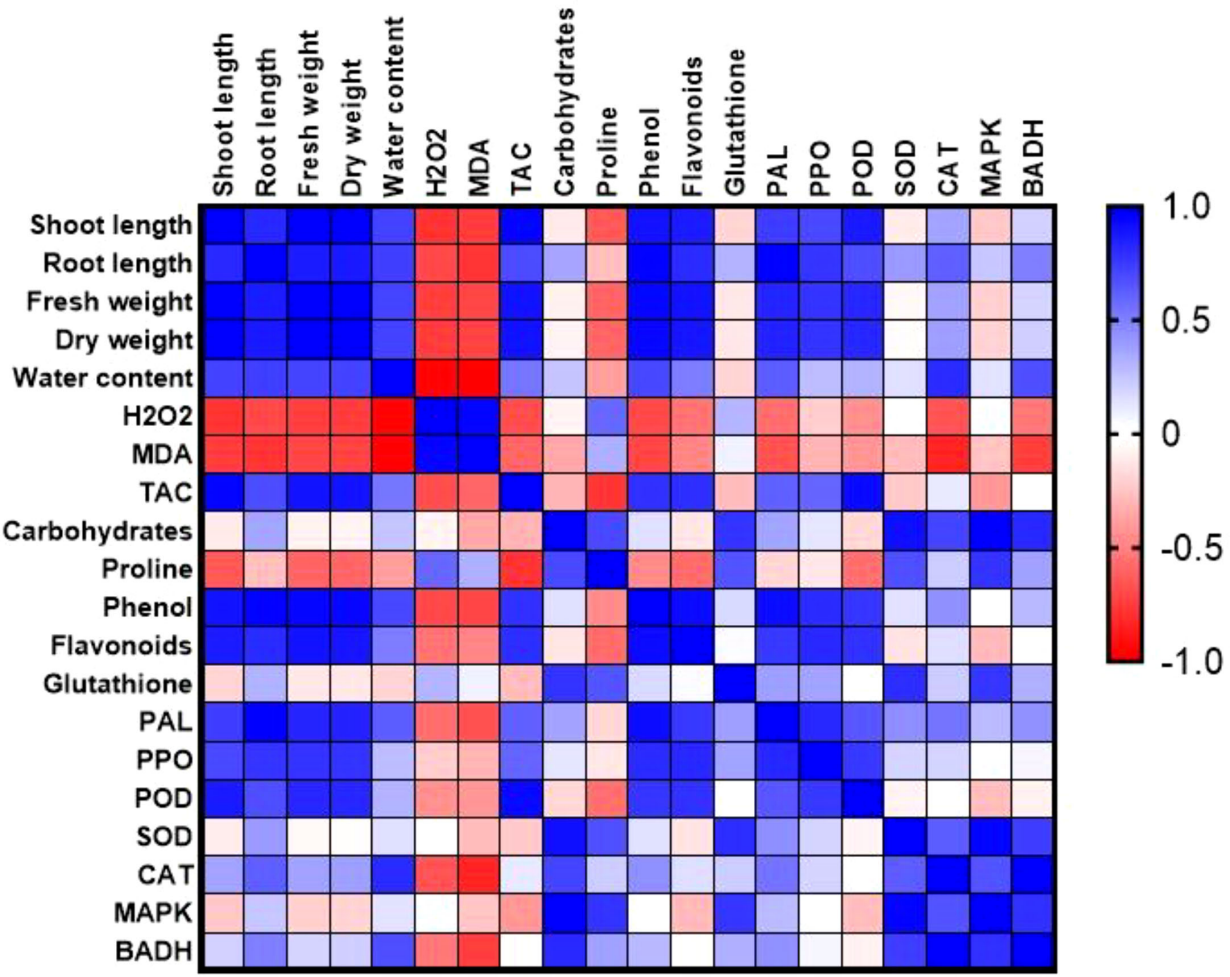
Correlation heatmap illustrating the relationships between morphological, physiological, and molecular parameters. The analysis is based on three biological replicates per treatment. Treatments included barley plants grown in 2023 treated with 25, 50 and 100 mg/L CaPNPs under control (well-watered) or (FC 30%) (drought stress). Color gradients indicate Pearson correlation coefficient ranging from +1.0 (strong positive; blue) to –1.0 (strong negative; red).

Pearson’s simple correlation provided a comprehensive visualization of the interrelationships among physiological, biochemical traits and molecular approaches in drought-stressed plants and treated with various concentrations of biogenic CaPNPs (**Figure 10**). Strong positive correlations (r > 0.9) were observed among growth parameters (shoot and root length, fresh and dry weight) and WC, indicating that improved hydration status directly supported biomass accumulation. These growth-related traits were also positively correlated (r > 0.85) with phenolic and flavonoid contents, as well as TAC, suggesting that enhanced secondary metabolism and antioxidant potential contributed to maintaining growth under stress. Growth parameters and WC were further associated with elevated activities of enzymatic antioxidants (PAL, PPO, POD, SOD, and CAT), reflecting a coordinated defense strategy in which increased antioxidant enzyme activities mitigate oxidative stress, thereby sustaining plant performance. In addition, expression levels of *HvMAPK3* and *HvBADH1* showed strong positive correlations (r > 0.8) with antioxidant enzymes, carbohydrates, Pro, and GSH, highlighting the regulatory role of these stress-responsive genes in modulating osmolyte accumulation and redox homeostasis. Conversely, oxidative stress markers (H_2_O_2_ and MDA) exhibited strong negative correlations (r < -0.85) with growth traits and antioxidants, confirming that higher oxidative damage impaired plant development and reduced metabolic efficiency. Interestingly, H_2_O_2_ and MDA showed positive correlations with Pro accumulation, which may reflect a compensatory response, where osmolyte build-up is triggered under conditions of oxidative stress to protect cellular functions.

## 4 Discussion

Water stress adversely affects various traits in barley, including its ability to germinate, the growth of the plumule and radicle, the development of roots and shoots, the rate of germination, and its capacity to absorb water (Bello et al., 2022). During drought stress in croplands, the lack of soil moisture leads to water depletion in deeper soil layers. As a result, overall plant biomass decreases, while the proportion of below-ground biomass (roots) increases relative to above-ground biomass (Ge et al., 2012). In accordance with this, an inhibition in shoot length and root length was recorded in our research in barley plants as a response to drought stress. Our results supported Bello et al. (2022) where excessive stress inhibits the length of roots in Malt Barley cultivars, due to dry soil conditions or reduced turgor, which is sufficient to limit cell elongation. Furthermore, drought stress led to decreases in both the fresh and dry weights of the plant, likely due to reduced cell division and growth caused by low turgor pressure under water-limited conditions (Ahluwalia et al., 2021). Biomass can be utilized to determine the potential productivity of the species under drought stress when evaluating its tolerance to drought (Sherstneva et al., 2024). In comparison with the control, water-stressed plants had lower WC, primarily due to impaired water uptake caused by low soil water potential under drought conditions. This water stress triggers an accumulation of abscisic acid (ABA), which leads to stomatal closure, a mechanism to reduce transpiration and prevent further water loss. This also led to reduced photosynthetic activity and water potential, which in turn caused a decline in biomass and a slower growth rate (Qiao et al., 2024). Ferioun et al. (2023) supported this by observing the detrimental effects of drought on water relations, physiological and biochemical processes, growth, and yield in barley. In our study, drought-stressed plants treated with CaPNPs were able to postpone dehydration effects and maintain tolerance by increasing shoot length and root length compared to drought treatment alone. As Pokhrel et al. (2025) explained that the resistance to drought might be influenced by their capacity to maintain a healthy root system under conditions of water stress.

LPO produces malondialdehyde, which is a biomarker for evaluating oxidative stress-related damage to organelles and plasmalemma membranes (Ayala et al., 2014). In *Brassica napus* plants, Ayyaz et al. (2022) observed that the administration of CaPNPs decreased oxidative stress damage as indicated by a decrease in ROS formation (hydrogen peroxide) or oxidative stress byproduct (malondialdehyde). In line with these findings, the present study observed a significant increase in MDA content in leaves of barley plants exposed to drought, and a notable reduction in MDA levels in drought-stressed plants sprayed with CaPNPs. It’s important to note that MDA is an indirect indicator of LPO, which in turn suggests potential membrane damage (Zhang et al., 2021). Moreover, Mazhar et al. (2023) reported lower levels of LPO in drought-affected leaves treated with various CaPNP doses, suggesting enhanced protection against oxidative stress during water deficit conditions. This reduction in LPO markers implies improved membrane integrity in response to CaPNPs application, as cellular membranes are often primary targets of various abiotic stresses (Kameniarová et al., 2022). Furthermore, calcium supplementation preserved membrane function and improved the integrity of cell membrane structure by combining with phospholipid molecules in the thylakoid membrane to generate calcium salts (Betterle et al., 2015). Wang et al. (2022) emphasized that one of the most significant physiological indicators of protoplasmic tolerance in plants is the ability of membranes to maintain their physical-chemical integrity under drought stress. While our MDA measurements provide valuable insights into the potential protective effects of CaPNPs against drought-induced oxidative damage, it’s crucial to interpret these results cautiously, recognizing that they offer an indirect estimation of membrane damage rather than a direct measurement (Zhang et al., 2021).

Under drought conditions, CaPNPs have demonstrated promising benefits on barley plants as indicated by enhancing the total antioxidant capacity in the present study. Research revealed that these nanoparticles enhance the overall antioxidant potential of the plants, an essential function in reducing drought-induced oxidative stress. For example, studies on finger millet (*Eleusine coracana*) showed that different plant growth parameters were greatly increased, and plant defense enzymes were activated by urea-doped calcium phosphate nanoparticles (CaP-U NPs) under both drought and irrigated conditions (Mishra et al., 2023). Furthermore, Ragab et al. (2025) revealed a high abundance of amino acids, minerals, phytohormones, and fatty acids in *J. rubens* extract which represents a promising and innovative biogenic approach for improving plant tolerance against abiotic stresses, owing to its nutritional and antioxidant characteristics.

It is well recognized that plants may tolerate drought stress by producing and storing soluble substances such as polyols, carbohydrates, betaines, and Pro (Kosar et al., 2021; Wang et al., 2024). It has been reported that by enhancing osmotic adjustment, protein stabilization, ROS detoxification, and cell membrane protection, these suitable solutes may help wheat plants to tolerate stress (Poudel, 2023). As reported by Ahmed et al. (2021), NPs can enhance stress tolerance against oxidative damage by increasing antioxidant activity, in addition to raising the concentration of osmolytes as soluble sugars, which support osmotic adjustment under drought stress conditions. In our research, osmolytes, especially Pro, were found to proliferate under drought stress. Several studies have demonstrated a beneficial relationship between the production of osmolytes and stress tolerance (Ramli et al., 2019). In agreement with our results, Khan et al. (2025) demonstrated that when maize plants exposed to water stress, Pro levels significantly promoted. The data showed that these compatible solutes function both as osmolytes and as protectors of macromolecules, helping to maintain protein folding and membrane stability under stress (Khan et al., 2023). The addition of CaPNPs resulted in insignificant rise in Pro levels, which agreed with results by Ayyaz et al. (2022) who reported the same effects in *Brassica napus* plants. Besides, barley plants stored more soluble sugars when CaPNPs were applied. Such increased osmolyte buildup reduced the osmotic potential of the cells, which allowed water to diffuse into the cell and maintain a higher turgor potential. Under water-limited conditions, plants can continue to perform physiological processes such as stomatal opening, CO_2_ absorption, and cell growth and development by maintaining a favorable cellular turgor potential (Eswaran et al., 2024).

Our results showed a decline in total flavonoid content (TFC) and total phenolic content (TPC) under drought stress, but an increase with CaPNPs application. This aligns with findings of Ayyaz et al. (2022) who reported that the application of Ca-NPs under drought conditions was associated with increased accumulation of secondary metabolites in *Brassica napus*, which may help counteract ROS-triggered damage. Khalil et al. (2019) reported that plants treated with Ca-NPs concurrently exhibited higher expression of secondary metabolites and an increased capacity to scavenge ROS. Our results are similar to previous studies on *Zataria multiflora* (Mosavat et al., 2019), which demonstrated that ZnO nanoparticles enhanced the expression of secondary metabolites.

GSH acts as an antioxidant by directly scavenging reactive oxygen species (ROS), such as ascorbate, or indirectly acting as a reducing agent to change ascorbic acid from its oxidized to its reduced form (Cruz de Carvalho, 2008). Drought enhanced GSH production in barley plants and this was in accordance with Abid et al. (2018), who reported an increase in GSH concentration in the sensitive cultivar of wheat under drought stress. The application of CaPNPs at 25 mg/L led to an increase in GSH accumulation. This suggests that at this concentration, plants primarily relied on GSH to combat oxidative stress. The impact of calcium nanoparticles on GSH levels varies according to the specific conditions, including nanoparticle concentration and the presence of additional cellular stresses such as drought exposure. At higher CaPNP concentrations (50 and 100 mg/L), the pattern of antioxidant response appeared to shift under water stress conditions. Such elevation in nanoparticle concentrations may trigger oxidative stress compared to lower dose, leading to a reduction in intracellular GSH levels as it is consumed in scavenging more ROS. Our results supported this explanation where the levels of H_2_O_2_ are provoked by increasing CaPNPs doses under drought conditions.

Measuring antioxidant enzyme activities during water stress treatments is one approach to assess the role of the scavenging system during drought stress. Successful adaptation to drought may rely on a plant’s capacity to sustain high antioxidants levels and prevent LPO under water deficit (Fotelli et al., 2002). Therefore, the level of damage to crops under water stress is determined by measuring the accumulated amount of MDA (Zhang et al., 2021). As confirmation, in the present work, higher constitutive and induced activities of PPO and POD was observed, in addition to *SOD* expression genes along with lower PAL activity and *CAT* gene under water stress. High *SOD* expression level shields the plant from superoxide radicles via activating SOD enzyme, but as it also changes O_2_ into H_2_O_2_, it cannot be regarded exclusively accountable for membrane protection against peroxidation. After that, additional enzymes such as CAT and POD should scavenge ROS to eliminate the H_2_O_2_ that SOD and other processes make (Knüver et al., 2025). In muskmelon genotypes, POD activity was found to be higher under conditions of water stress (Ansari et al., 2018). Furthermore, as reported by Iqbal and Yaning (2024), PPO and POD activity is probably an adaptive characteristic that could assist in overcoming tissue metabolic damage by lowering toxic levels of hydrogen peroxide generated during cell metabolism. POD is considered to play a role in several plant processes like oxidation of phenolics (Huo et al., 2024), regulation of cell elongation (Jeong et al., 2022) and detoxification of toxic compounds such as H_2_O_2_, which are produced due to oxidative stress (Stancill and Corbett, 2023).

To ensure the reproducibility and reliability of the data, a biological repetition across two different growing seasons was conducted, giving almost consistent results which reflect similar trends between treatments. This confirms the strong potential of calcium phosphate nanoparticles in mitigating drought-induced stress. Nevertheless, some analysis of the data is somewhat different between the two consecutive seasons even under similar growth conditions. Such variation may arise from slight microenvironmental differences (light, humidity, temperature), seed batch variability, changes in soil or microbial composition, or seasonal adaptation effects (Poorter and Garnier, 1999; Sadras and Richards, 2014).

It is noteworthy that drought-induced *HvSOD1* expression level in the leaves was accompanied by an increase in POD activity. Thus, our results suggested that POD activity coordinated with *HvSOD* genes as also proved by Thippeswamy et al. (2021) and the active involvement of them is related, at least in part, to drought-induced oxidative stress tolerance in barley plants treated with CaPNPs. Contrarily, our results revealed an intensive decline in *CAT1* genes and PAL activity under drought stress, with reference to normal conditions, but their activities were pronouncedly enhanced under the application of CaPNPs. This was in accordance with Abid et al. (2018) who recorded the decline in *HvCAT* expression level where the stress period prolonged to 10 days. The reason for this could be that these ROS scavengers are typically soluble in water and are eliminated either by self-oxidation or ROS detoxification. Under extended period of stress, the cell’s ability to resynthesize the oxidized or destroyed scavengers is limited (Khanna-Chopra and Selote, 2007). Consequently, with prolonged stress, tissues become particularly susceptible to ROS attack (Tripathy and Oelmüller, 2012).

CaPNPs have shown promising effects in enhancing plant tolerance to drought stress. Specifically, they can influence the activity of key enzymes and signaling pathways such as *Betaine Aldehyde Dehydrogenase* (*BADH1*) and *Mitogen-Activated Protein Kinases* (*MAPK3*). The production of GB, an osmoprotectant that assists plants in coping with osmotic stress during droughts, depends critically on BADH (Ayyaz et al., 2022). *MAPKs* phosphorylate various proteins, particularly transcription factors, to regulate the expression of genes involved in diverse cellular processes and stress responses. This phosphorylation cascade plays a pivotal role in modulating plant responses to drought and other environmental stresses (Zhang and Zhang, 2022; Zhao et al., 2023). It has been found that in *Arabidopsis thaliana*, drought stress activates *AtMKK1*, which subsequently causes the production of *AtMPK4* and controls stress-induced H_2_O_2_ through the catalase (CAT) pathway (Xing et al., 2007). Furthermore, abscisic acid (ABA) pathway is a key regulator of drought-responsive gene expression, including *MAPK* (Zhang et al., 2014). Thus, ABA-driven MAPK activation represents a conserved mechanism of drought tolerance. Our results showed an enhancement in MAPK pathway activation in barley, particularly under CaPNP treatment, reinforce and highlight CaPNPs as potent enhancers of ABA-mediated stress signaling. According to our results, drought diminished the level of expression of *BADH1*. This is incompatible with Wang et al. (2024) who stated that the synthesis of GB, which aids in protecting the photosynthetic apparatus of the plant and preserving water balance during droughts, can be increased by upregulating the expression of *BADH* genes. On the other hand, the application of CaPNPs boosted GB levels through higher BADH activity, contributing to the preservation of cellular structures and osmotic equilibrium. By stabilizing proteins and membranes, shielding the photosystem II complex, and lowering oxidative damage, this mechanism increases the plant’s resistance to drought (Niazian et al., 2021). The expression of *MAPK* is elevated in barley plants during drought stress (Zhou et al., 2022). The plant’s capacity to adapt to drought stress can be further enhanced by CaPNPs modifying the *MAPK3* signaling pathway. This modification helps in the activation of stress-responsive genes and proteins that contribute to improved drought tolerance (Ayyaz et al., 2022). Nonetheless, drought is a crucial hurdle that restricts crop yields, excessive researches of barley drought tolerance-related processes are critical for the genetic improvement of drought tolerance in this crop (Bapela et al., 2022). Since the use of nanoparticles in food products is done with great caution, the safe recommended dose in the environment and in plant products is up to and including 100 mg/L. In plants, Ayyaz et al. (2022) and Upadhyaya et al. (2017) emphasized the provoking role of 100 and 50 mg/L of CaPNPs in Brassica napus and rice, respectively. Regarding humans, Epple (2018) and Ha et al. (2015) pointed out the non-toxic and acceptable dose to be 100 mg/L or lower in blood cells and osteoblasts, in that order.

Taken together, after interpreting all the results and linking them, it can be said that CaPNPs were able to resist drought through regulating the buildup of osmoprotectants, enhancing antioxidant activity and influencing the expression of genes involved in drought response. The data strongly suggests that the lower dose (25 mg/L) is more beneficial under drought stress, while higher doses may be better for well-watered conditions. Such dose-dependent plant responses are linked to physiological state under drought versus control conditions. The focus on the 25 mg/L dose of CaPNPs is justified by its optimal effect under drought stress, as this low dose is sufficient to prime defense responses such as antioxidant enzyme activation and osmolyte accumulation without imposing metabolic burden or toxicity that higher doses might cause under stress. This dose likely hits a signaling threshold that efficiently triggers protective mechanisms like enhanced water retention and photosynthetic activity, mediated by key phytohormones such as abscisic acid as suggested by Li et al. (2017). Under well-watered control conditions, higher doses (50 and 100 mg/L) can support greater biomass accumulation and metabolic activity, because resource availability and stress signaling thresholds differ, making those doses more beneficial when stress is absent. Thus, the dose-dependent effect reflects the balance between inducing stress tolerance pathways without overwhelming cellular metabolism in drought, versus maximizing growth potential under optimal conditions. This nuanced interpretation enriches the discussion by linking nanoparticle dose, plant physiological state, and stress signaling dynamics in a sophisticated manner.

**Figure 11:**
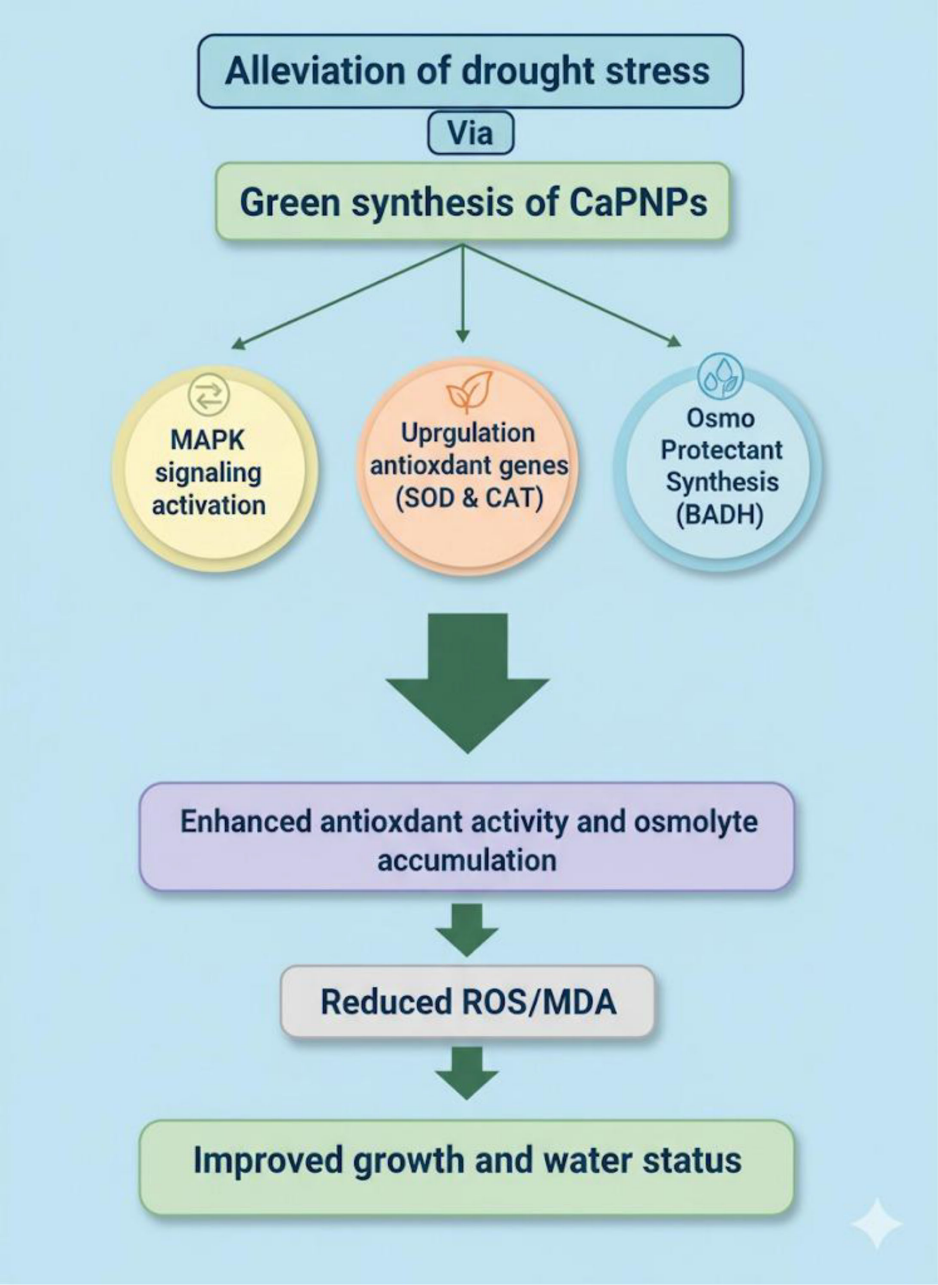
A schematic representation showing how plants treated with CaPNPs perceive drought stress through investigating physiological, biochemical and molecular approaches. The impact of CaPNPs in scavenging ROS, enhancing antioxidant activity and hence maintaining water status, regulates several functional features of plants such as activation of MAPK signaling, upregulation of antioxidant genes (*HvSOD1* and *HvCAT1*), besides accumulation of osmoprotectants (BADH).

Collectively and concisely, the exciting potential of biogenic CaPNPs under drought stress through revelation into metabolic, antioxidative and molecular approaches are highlighted in **Figure 11**. CaPNPs do not simply mitigate drought symptoms but activate a coordinated defense mechanism. They enhance antioxidant capacity by upregulating *HvSOD1*, *HvCAT1*, and POD activity, thereby improving ROS detoxification and membrane stability. Simultaneously, they promote osmotic balance through increased soluble sugars and BADH activity. The induction of stress-related genes, including *HvMAPK3*, highlights their role in transcriptional regulation. Together, this molecular evidence, together with the observed enhancement in antioxidant enzyme activities and osmolyte accumulation, supports that CaPNPs orchestrate a coordinated defense mechanism involving both ROS detoxification and osmotic regulation under drought conditions. Nevertheless, further molecular and mechanistic investigations (e.g., transcriptomic or proteomic analyses) are required to fully elucidate the underlying pathways.

## 5 Conclusion

The current study evaluated the possible function of biogenic CaPNPs in ameliorating oxidative damage caused by drought stress in *Hordeum vulgare* L. seedlings. Drought considerably hindered the growth of plants due to altered water conditions through increased ROS production. Our results demonstrated that the application of biogenic CaPNPs significantly enhanced the biochemical or physiological and molecular processes of the plant, including the maintenance of WC, the accumulation of osmoregulators, the activation of non-enzymatic as well as enzymatic antioxidants, in addition to molecular genes. The most effectual dose was 25 mg/L in drought conditions, whilst 50 and 100 mg/L at normal ones.

## Data Availability

The datasets presented in this study can be found in online repositories. The names of the repository/repositories and accession number(s) can be found in the article/**Supplementary Material**.

## References

[B1] Abid M. Ali S. Qi L. K. Zahoor R. Tian Z. Jiang D. . (2018). Physiological and biochemical changes during drought and recovery periods at tillering and jointing stages in wheat (Triticum aestivum L.). Sci. Rep. 8, 461–465. doi: 10.1038/s41598-018-21441-7, PMID: 29545536 PMC5854670

[B2] Ahluwalia O Singh P. C. Bhatia R. (2021). A review on drought stress in plants: Implications, mitigation and the role of plant growth promoting rhizobacteria. Resources, Environment and Sustainability 5, 100032.

[B3] Ahmed T. Noman M. Manzoor N. Shahid M. Abdullah M. Ali L. . (2021). Nanoparticle-based amelioration of drought stress and cadmium toxicity in rice via triggering the stress responsive genetic mechanisms and nutrient acquisition. Ecotoxicol Env. Saf. 209, 111–120. doi: 10.1016/j.ecoenv.2020.111829, PMID: 33383335

[B4] Ainsworth E. A. Gillespie K. M. (2007). Estimation of total phenolic content and other oxidation substrates in plant tissues using Folin–Ciocalteu reagent. Nat. Protoc. 2, 875–877. doi: 10.1038/nprot.2007.102, PMID: 17446889

[B5] Alabdallah N. M. Hasan M. M. Hammami I. Alghamdi A. I. Alshehri D. Alatawi H. A. (2021). Green synthesized metal oxide nanoparticles mediate growth regulation and physiology of crop plants under drought stress. Plants 10, 1730. doi: 10.3390/plants10081730, PMID: 34451775 PMC8399390

[B6] Al-Khayri J. M. Rashmi R. Surya Ulhas R. Sudheer W. N. Banadka A. Nagella P. . (2023). The role of nanoparticles in response of plants to abiotic stress at physiological, biochemical, and molecular levels. Plants 12, 292. doi: 10.3390/plants12020292, PMID: 36679005 PMC9865530

[B7] Anderson M. E. (1985). Determination of glutathione and glutathione sulfide in biological samples. Methods Enzym. 113, 548–555. doi: 10.1016/S0076-6879(85)13073-9, PMID: 4088074

[B8] Ansari W. A. Atri N. Singh B. Kumar P. Pandey S. (2018). Morpho-physiological and biochemical responses of muskmelon genotypes to different degree of water deficit. Photosynthetica 56, 1019–1030. doi: 10.1007/s11099-018-0821-9

[B9] Ayala A Muñoz M. F. Argüelles S. (2014). Lipid peroxidation: production, metabolism, and signaling mechanisms of malondialdehyde and 4‐hydroxy‐2‐nonenal. Oxidative Medicine and Cellular Longevity 2014, 360438., PMID: 24999379 10.1155/2014/360438 PMC4066722

[B10] Aynalem H. G. Kitaw M. B. (2023). Determination of total phenolic, total flavonoid and antioxidant activities of the leaf extracts of capparis tomentosa (Gumero). J. Bioprocess. Biotech. 13, 572. doi: 10.37421/2155-9821.2023.13.572

[B11] Ayyaz A. Fang R. Ma J. Hannan F. Huang Q. Athar H.-R. . (2022). Calcium nanoparticles (Ca-NPs) improve drought stress tolerance in Brassica napus by modulating the photosystem II, nutrient acquisition and antioxidant performance. NanoImpact 28, 100423. doi: 10.1016/j.impact.2022.100423, PMID: 36084849

[B12] Azeez L. Lateef A. Adetoro R. O. Adeleke A. E. (2021). Responses of Moringa oleifera to alteration in soil properties induced by calcium nanoparticles (CaNPs) on mineral absorption, physiological indices and photosynthetic indicators. Beni-Suef Univ. J. Basic Appl. Sci. 10, 39. doi: 10.1186/s43088-021-00128-5

[B13] Badea A. Wijekoon C. (2021). Benefits of Barley Grain in Animal and Human Diets. IntechOpen. doi: 10.5772/intechopen.97053

[B14] Badihi L. Gerami M. Akbarinodeh D. Shokrzadeh M. Ramezani M. (2021). Physio-chemical responses of exogenous calcium nanoparticle and putrescine polyamine in Saffron (Crocus sativus L.). Physiol. Mol. Biol. Plants 27, 119–133. doi: 10.1007/s12298-020-00923-x, PMID: 33627967 PMC7873192

[B15] Bapela T. Shimelis H. Tsilo T. J. Mathew I. (2022). Genetic improvement of wheat for drought tolerance: Progress, challenges and opportunities. Plants 11, 1331. doi: 10.3390/plants11101331, PMID: 35631756 PMC9144332

[B16] Bates L. S. Waldren R. P. A. Teare I. D. (1973). Rapid determination of free proline for water-stress studies. Plant Soil 39, 205–207. doi: 10.1007/BF00018060

[B17] Bello Z. A. Van Rensburg L. D. Dlamini P. Tfwala C. M. Tesfuhuney W. (2022). Characterisation and effects of different levels of water stress at different growth stages in malt barley under water-limited conditions. Plants 11, 578., PMID: 35270059 10.3390/plants11050578 PMC8912336

[B18] Betterle N. Ballottari M. Baginsky S. Bassi R. (2015). High light-dependent phosphorylation of photosystem II inner antenna CP29 in monocots is STN7 independent and enhances nonphotochemical quenching. Plant Physiol. 167, 457–471. doi: 10.1104/pp.114.252379, PMID: 25501945 PMC4326754

[B19] Carmona Fernández F. J. (2022). Nanosized calcium phosphates as novel macronutrient nano-fertilizers. Nanomaterials. 12 (15), 2709. doi: 10.3390/nano12152709, PMID: 35957141 PMC9370389

[B20] Cruz de Carvalho M. H. (2008). Drought stress and reactive oxygen species: production, scavenging and signaling. Plant Signal Behav. 3, 156–165. doi: 10.4161/psb.3.3.5536, PMID: 19513210 PMC2634109

[B21] Duan H. Liu W. Zhou L. Han B. Huo S. El-Sheekh M. . (2023). Improving saline-alkali soil and promoting wheat growth by co-applying potassium-solubilizing bacteria and cyanobacteria produced from brewery wastewater. Front. Environ. Sci. 11. doi: 10.3389/fenvs.2023.1170734

[B22] Eckstein D. Künzel V. Schäfer L. (2021). The global climate risk index 2021 (Bonn: Germanwatch).

[B23] El-Esawy M. A. Elkhateeb E. A. Hassan A. M. Elsherif D. E. (2025). Nanoparticle innovations: impact of biogenic CaP nanoparticles in mitigating the adverse effects of excessive nitrate application. Plant Soil 513, 1101–1120. doi: 10.1007/s11104-025-07233-9

[B24] El-Ghany M. F. A. El-Kherbawy M. I. Abdel-Aal Y. A. El-Dek S. I. Abd El-Baky T. (2021). Comparative study between traditional and nano calcium phosphate fertilizers on growth and production of snap bean (Phaseolus vulgaris L.) plants. Nanomaterials 11, 2913. doi: 10.3390/nano11112913, PMID: 34835677 PMC8625305

[B25] Elshaboury N. AlMetwaly W. M. (2023). Modeling construction and demolition waste quantities in Tanta City, Egypt: a synergistic approach of remote sensing, geographic information system, and hybrid fuzzy neural networks. Environ. Sci. pollut. Res. 30, 106533–106548. doi: 10.1007/s11356-023-29735-8, PMID: 37726636 PMC10579165

[B26] Epple M. (2018). Review of potential health risks associated with nanoscopic calcium phosphate. Acta Biomater. 77, 1–14. doi: 10.1016/j.actbio.2018.07.036, PMID: 30031162

[B27] Eswaran S. U. D. Sundaram L. Perveen K. Bukhari N. A. Sayyed R. Z. (2024). Osmolyte-producing microbial biostimulants regulate the growth of Arachis hypogaea L. under drought stress. BMC Microbiol. 24, 165. doi: 10.1186/s12866-024-03320-6, PMID: 38745279 PMC11094965

[B28] Ferioun M. Srhiouar N. Bouhraoua S. El Ghachtouli N. Louahlia S. (2023). Physiological and biochemical changes in Moroccan barley (Hordeum vulgare L.) cultivars submitted to drought stress. Heliyon 9. 10.1016/j.heliyon.2023.e13643 PMC9975271 36873157

[B29] Fotelli M. N. Rennenberg H. Gessler A. (2002). Effects of drought on the competitive interference of an early successional species (Rubus fruticosus) on Fagus sylvatica L. seedlings: 15N uptake and partitioning, responses of amino acids and other N compounds. Plant Biol. 4, 311–320. doi: 10.1055/s-2002-32334

[B30] Ge T. Sui F. Bai L. Tong C. Sun N. (2012). Effects of water stress on growth, biomass partitioning, and water-use efficiency in summer maize (Zea mays L.) throughout the growth cycle. Acta Physiol. Plant 34, 1043–1053. doi: 10.1007/s11738-011-0901-y

[B31] Ha S.-W. Jang H. L. Nam K. T. Beck G. R. Jr. (2015). Nano-hydroxyapatite modulates osteoblast lineage commitment by stimulation of DNA methylation and regulation of gene expression. Biomaterials 65, 32–42. doi: 10.1016/j.biomaterials.2015.06.039, PMID: 26141836 PMC4508253

[B32] Hayat F. Khanum F. Li J. Iqbal S. Khan U. Javed H. U. . (2023). Nanoparticles and their potential role in plant adaptation to abiotic stress in horticultural crops: A review. Sci. Hortic. 321, 112–120. doi: 10.1016/j.scienta.2023.112285

[B33] Heath R. L. Packer L. (1968). Photoperoxidation in isolated chloroplasts: I. Kinetics and stoichiometry of fatty acid peroxidation. Arch. Biochem. Biophys. 125, 189–198. doi: 10.1016/0003-9861(68)90654-1, PMID: 5655425

[B34] Huo Y. Li M. Wang X. Sun J. Zhou Y. Ma Y. . (2024). Rapid oxidation of phenolic compounds by O3 and HO: effects of the air–water interface and mineral dust in tropospheric chemical processes. Atmos. Chem. Phys. 24, 12409–12423. doi: 10.5194/acp-24-12409-2024

[B35] Iqbal H. Yaning C. (2024). Redox priming could be an appropriate technique to minimize drought-induced adversities in quinoa. Front. Plant Sci. 15, 1253677. doi: 10.3389/fpls.2024.1253677, PMID: 38638353 PMC11025396

[B36] Jeong Y. J. Kim Y.-C. Lee J. S. Kim D.-G. Lee J. H. (2022). Reduced expression of PRX2/ATPRX1, PRX8, PRX35, and PRX73 affects cell elongation, vegetative growth, and vasculature structures in Arabidopsis thaliana. Plants 11, 3353. doi: 10.3390/plants11233353, PMID: 36501391 PMC9740967

[B37] Jin X. Shi C. Yu C. Y. Yamada T. Sacks E. J. (2017). Determination of leaf water content by visible and near-infrared spectrometry and multivariate calibration in Miscanthus. Front. Plant Sci. 8, 721., PMID: 28579992 10.3389/fpls.2017.00721 PMC5437372

[B38] Kameniarová M. Černý M. Novák J. Ondrisková V. Hrušková L. Berka M. . (2022). Light quality modulates plant cold response and freezing tolerance. Front. Plant Sci. 13, 887103. doi: 10.3389/fpls.2022.887103, PMID: 35755673 PMC9221075

[B39] Khalil I. Yehye W. A. Etxeberria A. E. Alhadi A. A. Dezfooli S. M. Julkapli N. B. M. . (2019). Nanoantioxidants: Recent trends in antioxidant delivery applications. Antioxid 9, 24–30. doi: 10.3390/antiox9010024, PMID: 31888023 PMC7022483

[B40] Khan S. Siraj S. Shahid M. Haque M. M. Islam A. (2023). Osmolytes: Wonder molecules to combat protein misfolding against stress conditions. Int J Biol Macromol 234, 10–1016., PMID: 36796566 10.1016/j.ijbiomac.2023.123662

[B41] Khan P. Abdelbacki A. M. M. Albaqami M. Jan R. Kim K.-M. (2025). Proline promotes drought tolerance in maize. Biol. (Basel). 14, 41. doi: 10.3390/biology14010041, PMID: 39857272 PMC11762158

[B42] Khanna-Chopra R. Selote D. S. (2007). Acclimation to drought stress generates oxidative stress tolerance in drought-resistant than-susceptible wheat cultivar under field conditions. Env. Exp. Bot. 60, 276–283. doi: 10.1016/j.envexpbot.2006.11.004

[B43] Knüver T. Bär A. Hamann E. Zuber M. Mayr S. Beikircher B. . (2025). Stress dose explains drought recovery in Norway spruce. Front. Plant Sci. 16, 1542301. doi: 10.3389/fpls.2025.1542301, PMID: 40115942 PMC11922940

[B44] Kosar F. Akram N. A. Ashraf M. Ahmad A. AlYemeni M. N. Ahmad P. (2021). Impact of exogenously applied trehalose on leaf biochemistry, achene yield and oil composition of sunflower under drought stress. Physiol. Plant 172, 317–333. doi: 10.1111/ppl.13155, PMID: 32562257

[B45] Kumar K. B. Khan P. A. (1982). Effect of insecticides, oxydementon-methyl & dimethoate, on chlorophyll retention & hydrogen peroxide utilization in ragi (Eleusine coracana Gaertn. cv PR 202) leaves during senescence. Indian J. Exp. Biol. 20, 889–893., PMID: 7183525

[B46] Li K. Yang F. Zhang G. Song S. Li Y. Ren D. . (2017). AIK1, a mitogen-activated protein kinase, modulates abscisic acid responses through the MKK5-MPK6 kinase cascade. Plant Physiol. 173, 1391–1408. doi: 10.1104/pp.16.01386, PMID: 27913741 PMC5291029

[B47] Livak K. J. Schmittgen T. D. (2001). Analysis of relative gene expression data using real-time quantitative PCR and the 2– ΔΔCT method. Methods 25, 402–408. doi: 10.1006/meth.2001.1262, PMID: 11846609

[B48] Mansour E. Abdul-Hamid M. I. Yasin M. T. Qabil N. Attia A. (2017). Identifying drought-tolerant genotypes of barley and their responses to various irrigation levels in a Mediterranean environment. Agric. Water Manage. 194, 58–67. doi: 10.1016/j.agwat.2017.08.021

[B49] Masuko T. Minami A. Iwasaki N. Majima T. Nishimura S.-I. Lee Y. C. (2005). Carbohydrate analysis by a phenol–sulfuric acid method in microplate format. Anal. Biochem. 339, 69–72. doi: 10.1016/j.ab.2004.12.001, PMID: 15766712

[B50] Mazhar M. W. Ishtiaq M. Maqbool M. Hussain S. A. Casini R. Abd-ElGawad A. M. (2023). Seed nano-priming with calcium oxide maintains the redox state by boosting the antioxidant defense system in water-stressed carom (Trachyspermum ammi L.) plants to confer drought tolerance. Nanomaterials 13, 1453., PMID: 37176998 10.3390/nano13091453 PMC10180095

[B51] Meier S. Moore F. Morales A. González M.-E. Seguel A. Meriño-Gergichevich C. . (2020). Synthesis of calcium borate nanoparticles and its use as a potential foliar fertilizer in lettuce (Lactuca sativa) and zucchini (Cucurbita pepo). Plant Physiol. Biochem. 151, 673–680. doi: 10.1016/j.plaphy.2020.04.025, PMID: 32353673

[B52] Mira M. M. Hill R. D. Stasolla C. (2016). Phytoglobins improve hypoxic root growth by alleviating apical meristem cell death. Plant Physiol. 172, 2044–2056. doi: 10.1104/pp.16.01150, PMID: 27702845 PMC5100795

[B53] Mishra D. Chitara M. K. Upadhayay V. K. Singh J. P. Chaturvedi P. (2023). Plant growth promoting potential of urea doped calcium phosphate nanoparticles in finger millet (Eleusine coracana (L.) Gaertn.) under drought stress. Front. Plant Sci. 14, 1137002. doi: 10.3389/fpls.2023.1137002, PMID: 37255562 PMC10225717

[B54] Mosavat N. Golkar P. Yousefifard M. Javed R. (2019). Modulation of callus growth and secondary metabolites in different Thymus species and Zataria multiflora micropropagated under ZnO nanoparticles stress. Appl. Biochem. Biotechnol. 66, 316–322. doi: 10.1002/bab.1727, PMID: 30648768

[B55] Naidu S. Pandey J. Mishra L. C. Chakraborty A. Roy A. Singh I. K. . (2023). Silicon nanoparticles: Synthesis, uptake and their role in mitigation of biotic stress. Ecotoxicol. Environ. Saf. 255, 114783. doi: 10.1016/j.ecoenv.2023.114783, PMID: 36963184

[B56] Niazian M. Sadat-Noori S. A. Tohidfar M. Mortazavian S. M. M. Sabbatini P. (2021). Betaine aldehyde dehydrogenase (BADH) vs. flavodoxin (Fld): Two important genes for enhancing plants stress tolerance and productivity. Front. Plant Sci. 12, 650215. doi: 10.3389/fpls.2021.650215, PMID: 33868350 PMC8047405

[B57] Osundinakin M. Keshinro O. Atoloye E. Adetunji O. Afariogun T. Adekoya I. (2025). Enhancing drought resistance in African yam bean (Sphenostylis stenocarpa (Hochst. ex A. Rich.) Harms) through silicon nanoparticle priming: A multi-accession study. Plant Nano Biol. 12, 12–53. doi: 10.1016/j.plana.2025.100166

[B58] Pokhrel S. Kharel P. Pandey S. Botton S. Nugraha G. T. Holbrook C. . (2025). Understanding the impacts of drought on peanuts (Arachis hypogaea L.): exploring physio-genetic mechanisms to develop drought-resilient peanut cultivars. Front. Genet. 15, 1492434. doi: 10.3389/fgene.2024.1492434, PMID: 39845184 PMC11750809

[B59] Poorter H. Garnier E. (1999). “ Ecological significance of inherent variation in relative growth rate and its components,” in Handbook of Functional Plant Ecology. Eds. Pugnaire F. I. Valladares F. (NewYork: CRC Press), 81–120.

[B60] Poudel R. (2023). Effects of drought stress on growth and yield parameters of Zea mays—a comprehensive review. Agribus Manag Dev. N 1, 72–75. doi: 10.26480/amdn.02.2023.72.75

[B61] Prieto P. Pineda M. Aguilar M. (1999). Spectrophotometric quantitation of antioxidant capacity through the formation of a phosphomolybdenum complex: specific application to the determination of vitamin E. Anal. Biochem. 269, 337–341. doi: 10.1006/abio.1999.4019, PMID: 10222007

[B62] Qiao M. Hong C. Jiao Y. Hou S. Gao H. (2024). Impacts of drought on photosynthesis in major food crops and the related mechanisms of plant responses to drought. Plants 13, 1808., PMID: 38999648 10.3390/plants13131808 PMC11243883

[B63] Ragab G. A. Nessem A. A. Elshobary M. E. Henjes J. Razzaky E. O. (2025). Unraveling the physiological and ultrastructural responses of wheat to combat cobalt stress and the protective role of Jania rubens related to antioxidant defense and cellular integrity. Front. Plant Sci. 16, 1621482. doi: 10.3389/fpls.2025.1621482, PMID: 40688686 PMC12271171

[B64] Ramli M. Rossani R. B. Nadia Y. Darmawan T. B. Febriani Saiful . (2019). “ Nanoparticle fabrication of calcium oxide (CaO) mediated by the extract of red dragon fruit peels (Hylocereus Polyrhizus) and its application as inorganic–anti-microorganism materials,” in IOP Conf Ser Mater Sci Eng ( IOP Publishing), 120–130.

[B65] Rasheed A. Li H. Tahir M. M. Mahmood A. Nawaz M. Shah A. N. . (2022a). The role of nanoparticles in plant biochemical, physiological, and molecular responses under drought stress: A review. Front. Plant Sci. 13, 976179. doi: 10.3389/fpls.2022.976179, PMID: 36507430 PMC9730289

[B66] Rasheed A. Mahmood A. Maqbool R. Albaqami M. Sher A. Sattar A. . (2022b). Key insights to develop drought-resilient soybean: A review. J. King Saud Univ Sci. 34, 102–110. doi: 10.1016/j.jksus.2022.102089

[B67] Rezagholi M. Fard J. R. Darvishzadeh R. (2025). Selenium nanoparticles mitigates drought stress in E. purpurea by enhancing morpho-physiological characteristics and gene expression related to the phenylpropanoid pathway. Ind. Crops Prod. 227, 120833. doi: 10.1016/j.indcrop.2025.120833

[B68] Şirin S. Aydaş S. B. Aslım B. (2016). Biochemical evaluation of phenylalanine ammonia lyase from endemic plant cyathobasis fruticulosa (Bunge) Aellen. for the dietary treatment of phenylketonuria. Food Technol. Biotechnol. 54, 296. doi: 10.17113/ftb.54.03.16.4519, PMID: 27956861 PMC5151209

[B69] Sadras V. O. Richards R. A. (2014). Improvement of crop yield in dry environments: benchmarks, levels of organization and the role of nitrogen. J. Exp. Bot. 65, 1981–1995. doi: 10.1093/jxb/eru061, PMID: 24638898

[B70] Samynathan R. Venkidasamy B. Ramya K. Muthuramalingam P. Shin H. Kumari P. S. . (2023). A recent update on the impact of nano-selenium on plant growth, metabolism, and stress tolerance. Plants 12, 853. doi: 10.3390/plants12040853, PMID: 36840201 PMC9964709

[B71] SAS Institute Inc. (2005).SAS/STAT User’s Guide, Version 9.04.01 (Cary, NC, USA: SAS Institute Inc.).

[B72] Shah S. M. D. M. Shabbir G. Malik S. I. Raja N. I. Shah Z. H. Rauf M. . (2022). Delineation of physiological, agronomic and genetic responses of different wheat genotypes under drought condition. Agronomy 12, 1056. doi: 10.3390/agronomy12051056

[B73] Sherstneva O. Abdullaev F. Kior D. Yudina L. Gromova E. Vodeneev V. (2024). Prediction of biomass accumulation and tolerance of wheat seedlings to drought and elevated temperatures using hyperspectral imaging. Front. Plant Sci. 15, 1344826. doi: 10.3389/fpls.2024.1344826, PMID: 38371404 PMC10869465

[B74] Stancill J. S. Corbett J. A. (2023). Hydrogen peroxide detoxification through the peroxiredoxin/thioredoxin antioxidant system: A look at the pancreatic β-cell oxidant defense. Vitamins and hormones ( Elsevier)45–66., PMID: 10.1016/bs.vh.2022.11.001 PMC10058777 36707143

[B75] Thippeswamy M. Rajasreelatha V. Haleshi C. Sudhakar C. (2021). Modulation of cell components and specific isoforms of antioxidant enzymes in safflower under water stress and recovery. J. Plant Biochem. Physiol. 17, 94–105.

[B76] Tripathy B. C. Oelmüller R. (2012). Reactive oxygen species generation and signaling in plants. Plant Signal Behav. 7, 1621–1633. doi: 10.4161/psb.22455, PMID: 23072988 PMC3578903

[B77] Upadhyaya H. Begum L. Dey B. Nath P. K. Panda S. K. (2017). Impact of calcium phosphate nanoparticles on rice plant. J. Plant Sci. Phytopathol. 1, 1–10. doi: 10.29328/journal.jpsp.1001001

[B78] Van Nguyen D. Nguyen H. M. Le N. T. Nguyen K. H. Nguyen H. T. Le H. M. . (2022). Copper nanoparticle application enhances plant growth and grain yield in maize under drought stress conditions. J. Plant Growth Regul. 41, 364–375. doi: 10.1007/s00344-021-10301-w

[B79] Velikova V. Yordanov I. Edreva A. (2000). Oxidative stress and some antioxidant systems in acid rain-treated bean plants: protective role of exogenous polyamines. Plant Sci. 151, 59–66. doi: 10.1016/S0168-9452(99)00197-1

[B80] Wang X. Li X. Zhao W. Hou X. Dong S. (2024). Current views of drought research: experimental methods, adaptation mechanisms and regulatory strategies. Front. Plant Sci. 15, 1371895. doi: 10.3389/fpls.2024.1371895, PMID: 38638344 PMC11024477

[B81] Wang J. Zhang X. Han Z. Feng H. Wang Y. Kang J. . (2022). Analysis of physiological indicators associated with drought tolerance in wheat under drought and re-watering conditions. Antioxidants 11, 2266. doi: 10.3390/antiox11112266, PMID: 36421452 PMC9687282

[B82] Woznicki S. A. Nejadhashemi A. P. Parsinejad M. (2015). Climate change and irrigation demand: Uncertainty and adaptation. J. Hydrol. Reg. Stud. 3, 247–264. doi: 10.1016/j.ejrh.2014.12.003

[B83] Xing Y. Jia W. Zhang J. (2007). AtMEK1 mediates stress-induced gene expression of CAT1 catalase by triggering H2O2 production in Arabidopsis. J. Exp. Bot. 58, 2969–2981. doi: 10.1093/jxb/erm144, PMID: 17728292

[B84] Ying Y. Q. Song L. L. Jacobs D. F. Mei L. Liu P. Jin S. H. . (2015). Physiological response to drought stress in Camptotheca acuminata seedlings from two provenances. Front. Plant Sci. 6, 361. doi: 10.3389/fpls.2015.00361, PMID: 26052334 PMC4440367

[B85] Zhang H. Liu Y. Wen F. Yao D. Wang L. Guo J. . (2014). A novel rice C2H2-type zinc finger protein, ZFP36, is a key player involved in abscisic acid-induced antioxidant defence and oxidative stress tolerance in rice. J. Exp. Bot. 65, 5795–5809. doi: 10.1093/jxb/eru313, PMID: 25071223 PMC4203119

[B86] Zhang Y. Luan Q. Jiang J. Li Y. (2021). Prediction and utilization of malondialdehyde in exotic pine under drought stress using near-infrared spectroscopy. Front. Plant Sci. 12, 735275. doi: 10.3389/fpls.2021.735275, PMID: 34733301 PMC8558207

[B87] Zhang M. Zhang S. (2022). Mitogen-activated protein kinase cascades in plant signaling. J. Integr. Plant Biol. 64, 301–341. doi: 10.1111/jipb.13215, PMID: 34984829

[B88] Zhao Y. Zheng X. Zhang X. Wang W. Cai G. Bi G. . (2023). PIF3 is phosphorylated by MAPK to modulate plant immunity. New Phytol. 240, 372–381. doi: 10.1111/nph.19139, PMID: 37475167

[B89] Zhou M. Zhao B. Li H. Ren W. Zhang Q. Liu Y. . (2022). Comprehensive analysis of MAPK cascade genes in sorghum (Sorghum bicolor L.) reveals SbMPK14 as a potential target for drought sensitivity regulation. Genom 114, 110–135. doi: 10.1016/j.ygeno.2022.110311, PMID: 35176445

