# Supplementary material for: The effect of biogenic calcium phosphate nanoparticles on drought-stressed barley
Source: Front Plant Sci. 2025 Dec 4;16:1660534. doi: 10.3389/fpls.2025.1660534 (PMC12711726; doi:10.3389/fpls.2025.1660534)
Supplement: Supplementary file 1 [file DataSheet1.docx]

**Table 1.** A list of the RT-PCR primers.

| ***Gene name*** | **Sequence (5’-3’)** |
| --- | --- |
| ***HvACTIN2-F*** | GGTAACATTGTGCTCAGTGGTGG |
| ***HvACTIN2-R*** | AACGACCTTAATCTTCATGCTGC |
| ***HvBADH1-F*** | ACTAGCGTCCGCAACAGCAG |
| ***HvBADH1-R*** | CAAAGCACCCAGCAACATCA |
| ***HvCAT1-F*** | ACTACGACGGGCTCATG |
| ***HvCAT1-R*** | GGAGCTGAGACGGCTTC |
| ***HvMAPK3-F*** | CTTTAACCCGCTGCAGAGGA |
| ***HvMAPK3-R*** | GTCAAAGGAGAAGGGGTCCG |
| ***HvSOD1-F*** | CACCTCCACCACCAACCCCCAAAAG |
| ***HvSOD1-R*** | AATGGCGTCGTTACAAGTATGACTG |

## Preliminary experiment results

### Growth Characteristics

###

**Supplemental Figure 1.** Growth parameters of barley plants treated with 25, 50 and 100 mg/L CaPNPs under control (well-watered) or (FC 30%) in 2022. A. Shoot length, B. Root length, C. Fresh weight, D. Dry weight, of barley plants under control and drought conditions with various CaPNP treatments. Values represent mean ± SD (n=3). Different letters indicate significant differences at p < 0.05 (Tukey’s ).

### Stress Biomarkers

**Supplemental Figure 2.** Effects of calcium phosphate nanoparticles (CaPNPs) on stress biomarkers in barley plants under well-watered (control) and drought stress (FC 30%) conditions in 2022. The figure presents the effects of various concentrations of CaPNPs (25, 50, and 100 mg/L) on two key stress indicators: A. H_2_O_2_ (Hydrogen Peroxide) and B. MDA (Malondialdehyde): The product of lipid peroxidation. Values represent mean ± SD (n=3). Different letters indicate significant differences at p < 0.05 (Tukey’s ).

### Total Antioxidant Activity

**Supplemental Figure 3.** Total antioxidant capacity (TAC) of barley plants treated with 25, 50 and 100 mg/L CaPNPs under control (well-watered) or (FC 30%) in 2022. Values represent mean ± SD (n=3). Different letters indicate significant differences at p < 0.05 (Tukey’s ).

### Osmoprotectants

**Supplemental Figure 4.** Osmolytes accumulation in barley plants treated with 25, 50 and 100 mg/L CaPNPs under control (well-watered) or (FC 30%) in 2022. A. Total carbohydrate and B. Proline. Values represent mean ± SD (n=3). Different letters indicate significant differences at p < 0.05 (Tukey’s ).

### Non-Enzymatic Antioxidant Molecules

**Supplemental Figure 5.** Non-enzymatic antioxidants of barley plants treated with 25, 50 and 100 mg/L CaPNPs under control (well-watered) or (FC 30%) in 2022. A. Total phenolic contents. B. Total Flavonoids, C. Reduced Glutathione. Values represent mean ± SD (n=3). Different letters indicate significant differences at p < 0.05 (Tukey’s ).

### Antioxidant Enzyme Activities

**Supplemental Figure 6.** Activities of antioxidant enzymes of barley plants treated with 25, 50 and 100 mg/L CaPNPs under control (well-watered) or (FC 30%) in 2022. Values represent mean ± SD (n=3). Different letters indicate significant differences at p < 0.05 (Tukey’s ).
